# “Thinking” vs. “Talking”: Differential Autocrine Inflammatory Networks in Isolated Primary Hepatic Stellate Cells and Hepatocytes under Hypoxic Stress

**DOI:** 10.3389/fphys.2017.01104

**Published:** 2017-12-22

**Authors:** Yoram Vodovotz, Richard L. Simmons, Chandrashekhar R. Gandhi, Derek Barclay, Bahiyyah S. Jefferson, Chao Huang, Rami Namas, Fayten el-Dehaibi, Qi Mi, Timothy R. Billiar, Ruben Zamora

**Affiliations:** ^1^Department of Surgery, University of Pittsburgh, Pittsburgh, PA, United States; ^2^Center for Inflammation and Regenerative Modeling, McGowan Institute for Regenerative Medicine, University of Pittsburgh, Pittsburgh, PA, United States; ^3^Pittsburgh Liver Research Center, University of Pittsburgh, Pittsburgh, PA, United States

**Keywords:** liver, hepatocyte, stellate cell, cytokines, systems biology, hypoxia

## Abstract

We hypothesized that isolated primary mouse hepatic stellate cells (HSC) and hepatocytes (HC) would elaborate different inflammatory responses to hypoxia with or without reoxygenation. We further hypothesized that intracellular information processing (“thinking”) differs from extracellular information transfer (“talking”) in each of these two liver cell types. Finally, we hypothesized that the complexity of these autocrine responses might only be defined in the absence of other non-parenchymal cells or trafficking leukocytes. Accordingly, we assayed 19 inflammatory mediators in the cell culture media (CCM) and whole cell lysates (WCLs) of HSC and HC during hypoxia with and without reoxygenation. We applied a unique set of statistical and data-driven modeling techniques including Two-Way ANOVA, hierarchical clustering, Principal Component Analysis (PCA) and Network Analysis to define the inflammatory responses of these isolated cells to stress. HSC, under hypoxic and reoxygenation stresses, both expressed and secreted larger quantities of nearly all inflammatory mediators as compared to HC. These differential responses allowed for segregation of HSC from HC by hierarchical clustering. PCA suggested, and network analysis supported, the hypothesis that above a certain threshold of cellular stress, the inflammatory response becomes focused on a limited number of functions in both HSC and HC, but with distinct characteristics in each cell type. Network analysis of separate extracellular and intracellular inflammatory responses, as well as analysis of the combined data, also suggested the presence of more complex inflammatory “talking” (but not “thinking”) networks in HSC than in HC. This combined network analysis also suggested an interplay between intracellular and extracellular mediators in HSC under more conditions than that observed in HC, though both cell types exhibited a qualitatively similar phenotype under hypoxia/reoxygenation. Our results thus suggest that a stepwise series of computational and statistical analyses may help decipher how cells respond to environmental stresses, both within the cell and in its secretory products, even in the absence of cooperation from other cells in the liver.

## Introduction

In settings of stress, such as liver ischemia/reperfusion, hemorrhagic shock after trauma, and drug-induced liver injury, endogenous mediators released from liver cells are known to initiate and regulate sterile liver inflammation (Peitzman et al., 1995; Vodovotz et al., 2004; Sun et al., 2017; Woolbright and Jaeschke, 2017). There are two major types of cells in the liver: parenchymal cells [hepatocytes (HC)] and non-parenchymal cells [sinusoidal endothelial cells, phagocytic Kupffer cells, and hepatic stellate cells (HSC)]. Whereas 70–85% of the normal adult murine liver volume is occupied by HC (Kmieć, 2001), Kupffer cells—which comprise approximately 35% of the non-parenchymal liver cells in normal adult mice (Bilzer et al., 2006)—play a central role in responses to early reperfusion injury following hypoxia/reoxygenation due to their macrophage-like properties (Bilzer and Gerbes, 2000). Studies over the past several decades have focused predominantly on the stress responses of HC, Kupffer cells (a heterogeneous cell population) and trafficking innate immune cells (de Groot, 1992; Peitzman et al., 1995; West and Wilson, 1996). Lesser attention has been paid to sinusoidal or vascular endothelial cells and HSC and the inherent autocrine complexity of the cellular components of the normal liver under stressful conditions have rarely been the subject of investigation.

Various studies have focused on the responses of HC cell lines to hypoxia/reoxygenation. For example, early studies showed that human HepG2 hepatoma cells express interferon-γ (IFNγ), Tumor Necrosis Factor-α (TNF-α), Transforming Growth Factor-β1 (TGFβ1), Macrophage Colony-Stimulating Factor (M-CSF), Oncostatin-M (OSM), Intercellular Adhesion Molecule (ICAM-1), Interleukin 4 (IL-4), IL-5, IL-7, IL-10, IL-11, IL-12, and IL-6 receptor (IL-6R), while the expression of IL-1β, IL-2, IL-3, IL-6, CD40 ligand and IL-2R genes was not detected (Stonans et al., 1999). Normal HC seem to have a much narrower spectrum of response: for example, primary human HC were shown to release IFNγ, IL-12p40, IL-12p70, IL-17A, and IL-10 following exposure to hepatotoxic drugs (Ogese et al., 2017). We recently used Luminex™ technology coupled with computational analyses to study the *in vitro* response of mouse HC to hypoxia (Ziraldo et al., 2013). We found that many inflammatory mediators were changed significantly in both normoxic and hypoxic cultures, and MCP-1 was identified as central node in the inflammatory networks of HC and as an inducer of IL-6; segregating trauma patients based on their co-expression of MCP-1 and IL-6 allowed us to suggest MCP-1 as a potential biomarker for clinical outcomes in trauma/hemorrhagic shock (Ziraldo et al., 2013).

HSC represent only 5-8% of the total number of liver cells (Geerts, 2001). The inflammatory responses of this cell type to hypoxia/reoxygenation are less well studied, though HSC are considered important in the pathogenesis of liver fibrosis (de Oliveira da Silva et al., 2017); furthermore, protection of the liver cells from ischemia/reperfusion injury in HSC-depleted mice indicates that HSC are major contributors to liver damage (Stewart et al., 2014). DNA microarray analyses have shown that hypoxia regulates the expression of genes that may alter the sensitivity of HSC to chemotaxins and other stimuli, and regulates the expression of genes important for angiogenesis and collagen synthesis (Copple et al., 2011). Furthermore, in primary HSC, bacterial lipopolysaccharide (LPS) strongly up-regulated numerous CC and CXC chemokines as well as IL-17F (Harvey et al., 2013). Other studies showed that HSC can express a number of other cytokines and chemokines such as Eotaxin, IFNγ, IL-6, IL-8, and IL-10 (Berardis et al., 2014).

Most studies of the effects of environmental stress are carried out *in vivo*, in which the interaction of various cell types within an organ cannot be discerned from one another. To our knowledge, no studies have utilized pure hepatic cell cultures to examine the autocrine effects, which themselves reflect complexity of response even in the absence of interaction with neighboring cell types. In addition, most studies explore the repertoire of mediator secretion (“talking”) by stressed cells, without questioning the internal reactions that take place within the cell (“thinking”). The interpretation of such complex responses require a baseline of information about the inherent responsiveness of purified cells. In addition, mere description of the secretory output of a cell under stress does not adequately address the complexity of even the simplest of experimental systems. For this reason, and given the close proximity of HSC and HC (one HSC contacting two HC on either side of the central vein), this study was designed to compare the intracellular and secretory (autocrine) responses of pure HC and HSC in culture to hypoxic stress and reoxygenation using a unique stepwise series of statistical and data driven modeling techniques previously used to define dynamic molecular networks in both experimental and clinical trauma and acute systemic inflammation (Mi et al., 2011; Ziraldo et al., 2011, 2013; Zaaqoq et al., 2014; Almahmoud et al., 2015; Abboud et al., 2016; Namas et al., 2016). These approaches, including Two-Way ANOVA, hierarchical clustering, Principal Component Analysis (PCA) and Network Analysis were chosen to unmask the complexity of even simple cellular systems. Our results suggest that intracellular inflammatory networks and principal characteristics (“thinking”) differ in several important respects from those in the conditioned medium (“talking”) of both HSC and HC. Our results further suggest that a stepwise series of computational and statistical analyses may help decipher how cells respond to inflammatory stresses.

## Materials and methods

### Materials

Williams Medium E, penicillin, streptomycin, L-glutamine, and HEPES were purchased from Invitrogen (Carlsbad, CA). Insulin (Humulin®) was purchased from Eli Lilly (Indianapolis, IN), and calf serum was obtained from HyClone Laboratories (Logan, UT). Tissue culture dishes were from Corning Glass Works (Corning, NY).

### Liver cell isolation and culture

This study was carried out in accordance with the recommendations of the National Institutes of Health. The protocol was approved by the Institutional Animal Care and Use Committee of the University of Pittsburgh. The liver cell isolation and culture procedures were as follows:

*-Primary Hepatocytes (HC)* were harvested from adult C57BL/6 male mice (Charles River Laboratories, Wilmington, MA). Cells were isolated by collagenase perfusion using the method of Seglen (1976) and purified to >98% purity by repeated centrifugation at 50 g, followed by further purification over 30% Percoll. Viability at time of plating was checked by trypan blue exclusion. Highly purified HC (>98% purity and >95% viability by trypan blue exclusion) were suspended in Williams' E medium supplemented with 10% heat-inactivated calf serum, 15 mM HEPES (pH 7.4), 16 units insulin, 2 mM L-glutamine, 100 units/ml penicillin, and 100 μg/ml streptomycin. The cells were then plated on collagen-coated cell culture dishes (3 × 10^6^ cells/6-cm dish) or plates (250,000 cells/well in 6-well plates) and cultured overnight at 37°C. The old medium was then removed and cells were further incubated with fresh media containing 5% heat-inactivated calf serum under 21% O_2_ (C, Control), 6 h hypoxia (H) or 6 h hypoxia followed by reoxygenation as previously described (Metukuri et al., 2009). Hypoxic conditions were obtained by placing the cells into a modular incubator chamber (Billups-Rothenburg, Del Mar, CA) flushed with a hypoxic gas mixture containing 1% O_2_, 5% CO_2_ and 94% N_2_. Duplicate hypoxic cultures were returned to 21% O_2_ for reoxygenation overnight (18 h). At the end of the experiments, both cell culture media (CCM) and whole cell lysate (WCL) were collected and stored at −80°C until analysis. Total protein isolation and determination was done using the BCA protein assay kit from Pierce (Rockford, IL) with bovine serum albumin as standard as previously described (Metukuri et al., 2009). All data were normalized and the final mediator concentrations were expressed in pg/mg total protein. For data analysis and unless otherwise indicated, the number of independent experiments (*n*) refers to the number of separate mice from which HC or HSC were harvested. Experimental data are presented as mean ± SEM.

*-Primary Hepatic Stellate Cells (HSC)* were isolated as previously described (Dangi et al., 2012). Briefly, mouse livers from C57BL/6 mice were perfused *in situ* through the inferior vena cava with 30–40 ml HBSS (without Ca^2+^), then digested with 30–40 ml HBSS (with Ca^2+^) containing collagenase type IV (0.25 mg/ml) (Worthington, Lakewood, NJ) and protease (0.50 mg/ml) (Sigma, St. Louis, MO). The cell suspension was filtered through 100 μm nylon mesh. Following removal of HC and cell debris by low speed centrifugation (50 g; 2 min), HSC were purified by Histodenz density gradient centrifugation, and suspended in DMEM containing 100 U/ml penicillin, 100 μg/ml streptomycin, 10% FBS and 10% horse serum. Cells were seeded in gelatin (0.1% in PBS)-coated plates at a density of 0.5 × 10^6^/cm^2^ and 20 min later, loosely adherent HSC were harvested and re-seeded in new 6 or 96-well flat-bottom plates. Cell purity, as assessed by vitamin A autofluorescence, desmin and glial fibrillary acidic protein (GFAP) immunostaining (Sumpter et al., 2012), was >98%. HSC were cultured under 21% O_2_, 6 h hypoxia or 6 h hypoxia followed by reoxygenation for 18 h, then harvested for protein isolation and analysis as described above for HC.

### Analysis of inflammatory mediators

Mouse inflammatory mediators were measured using a Luminex® 100 IS apparatus (Luminex, Austin, TX) and the BioSource 20-plex™mouse cytokine bead kit (BioSource-Invitrogen, San Diego, CA) as per manufacturer's specifications. The antibody bead kit included: Basic fibroblast growth factor (FGF basic), Granulocyte-Macrophage Colony-Stimulating Factor (GM-CSF), Interferon-γ (IFN-γ), Interleukin (IL)-1α, IL-1β, IL-2, IL-4, IL-5, IL-6, IL-10, IL-12 (both p40 and p70 subunits), IL-13, IL-17A, Interferon-γ-inducible Protein 10 (IP-10/CXCL10), Keratinocyte-derived Cytokine (KC/CXCL1), Monocyte Chemoattractant Protein-1 (MCP-1/CCL2), Monokine induced by Interferon-γ (MIG/CXCL9), Macrophage Inflammatory Protein-1α (MIP-1α/CCL3), Tumor Necrosis Factor-α (TNF-α), and Vascular Endothelial Growth Factor (VEGF).

### Statistical and computational analyses

**Figure 1:**
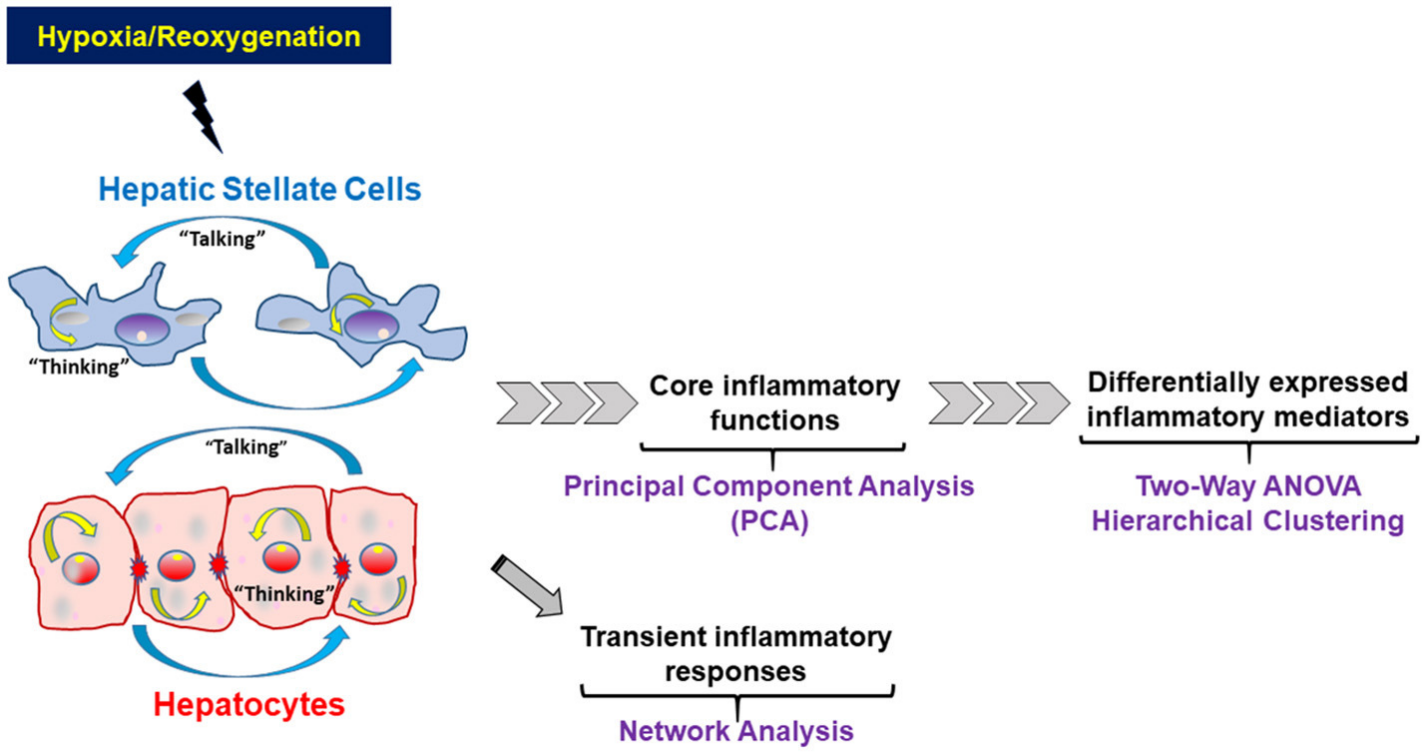
Schematic of the statistical and computational methods and the rationale for their use to study the way liver cells respond to inflammatory stimuli. Hepatocytes and Hepatic Stellate Cells respond to hypoxic stress and reoxygenation by both producing and releasing inflammatory mediators that form defined networks, which can be characterized using network analysis techniques. As the presence of signals and networks persists, early regulatory cytokines begin to be secreted. Some of these core mediators are present at low levels, often with high variance, and their presence and effect may be inferred, as an initial filter, using techniques such as Principal Component Analysis (PCA). At the same time, other inflammatory mediators are usually significantly elevated as defined by standard statistical analyses and thus can be defined as biomarkers of elevated inflammation.

We applied a series of statistical and data-driven modeling techniques aimed at discovering principal drivers, interconnected networks, and potential key regulatory nodes of inflammation in HSC vs. HC following increasing levels of cellular stress. We applied these methods in a stepwise fashion based on our concept of the way liver cells respond to inflammatory stimuli (Figure 1, adapted from Namas et al., 2015). Importantly, we also sought to address intracellular information processing (“thinking”) as well as extracellular inflammatory mediator production (“talking”) carried out by each cell type individually, as well as inferring how these two processes may interact in each cell type. In brief, we hypothesize that low-level stresses associated with cell culture, as well as more stressful stimuli such as hypoxia with or without reoxygenation, will cause liver cells to produce a panoply of inflammatory mediators in order to restore homeostasis. We hypothesize that this dynamic production of inflammatory mediators takes the form of inflammatory networks, and thus can be elucidated using network inference algorithms. As these early inflammatory responses progress, and in the face of continued hypoxia or the onset of reoxygenation, these networks coalesce around an intermediate, core set of inflammation-associated cellular functions, which we hypothesize can be inferred via PCA and related methods. These intermediate processes evolve to form a differential set of inflammatory responses characterized by distinct mediators that characterize the autocrine response of each liver cell type. These differential responses can be discerned both by standard statistical approaches such as Two-way ANOVA as well as data-driven modeling tools such as hierarchical clustering (Figure 1). Accordingly, we followed the stepwise series of analyses detailed below:

1. *Two-Way Analysis of Variance (ANOVA)* was carried out to analyze the changes in inflammatory mediators in HC vs. HSC in both CCM and WCL, using *SigmaPlot* (Systat Software, San Jose, CA) as indicated.
2. *Unsupervised Hierarchical Clustering* was carried out in an attempt to differentiate the ultimate inflammatory responses of HC and HSC based on concentrations of inflammatory mediators in both CCM and WCL during each of the three experimental conditions examined: control (6 h), hypoxia (6 h) and hypoxia/reoxygenation (6 h/18 h). This analysis was performed using mediator raw values using Matlab® software (The MathWorks, Inc., Natick, MA) as previously described (Ziraldo et al., 2013). Fold change values for each inflammatory mediator are represented in heat maps, ranging from large negative to large positive values as shown in the Figure Legends.
3. *Principal Component Analysis (PCA)* was carried out to identify those inflammatory mediators that were the most characteristic of the intermediate, multivariate response of each cell type (HC vs. HSC) and experimental condition (control, hypoxia, and hypoxia/reoxygenation) using Matlab® software. To perform this analysis, the data were first normalized for each inflammatory mediator (i.e., a given value divided by the maximum value for a given inflammatory mediator), so that all mediator levels were converted into the same scale (from 0 to 1). In this way, any artifactual effects on variance due to the different ranges of concentration observed for different cytokines were eliminated. Only sufficient components to capture at least 70% of the variance in the data were considered. From these leading principal components, the coefficient (weight) associated with each inflammatory mediator was multiplied by the eigenvalue associated with that principal component. This product represented the contribution of a given mediator to the variance accounted for in that principal component. The overall score given to each mediator is the sum of its scores in each component, depicted as a stacked bar graph. This gives a measure of a given inflammatory mediator's contribution to the overall variance of the system. The mediators with the largest scores are the ones which contributed most to the variance of the process being studied (Mi et al., 2011).
4. *Network Analysis* was carried out to define the central inflammatory network nodes as a function of cell type (i.e., HC vs. HSC), experimental condition, and spatial localization (lysate vs. conditioned medium) using a modified version of our previously published algorithm for Dynamic Network Analysis (Mi et al., 2011; Ziraldo et al., 2013; Zamora et al., 2016). Connections between inflammatory mediators were created if the Pearson correlation coefficient between any two nodes (inflammatory mediators) was greater or equal to a threshold of 0.95, as indicated. The “network complexity” for each experimental condition was calculated using the following formula: Sum (N_1_ + N_2_ +…+ N_n_)/n-1, where N represents the number of connections for each mediator and n is the total number of mediators analyzed. The total number of connections represents the sum of the number of connections in a given experimental sub-group.

## Results

In the present study, we sought to compare, in a systematic fashion, the inflammatory responses of freshly isolated HC or HSC to hypoxia and hypoxia/reoxygenation. We reasoned that distinct statistical and computational analyses would elucidate different steps in the intracellular and extracellular responses of these cells to stress, shown schematically in Figure 1 (see *Materials and Methods* for details). In the context of that schematic, we worked backwards from the most directly apparent/final responses (assessed by Two-Way ANOVA and hierarchical clustering) to the intermediate responses (assessed by PCA), and then to discerning the most proximal responses to stress (assessed by network analysis). Another key goal of our studies was to utilize computational modeling to assess the interactions between intracellular information processing (“thinking”) vs. extracellular information transfer (“talking”) in both HC and HSC. For that reason, we examined both the whole-cell lysates (WCL) and the cell culture media (CCM) in each cell type.

### Differential production and release of inflammatory mediators in HSC vs. HC in response to hypoxia and hypoxia/reoxygenation

**Figure 2:**
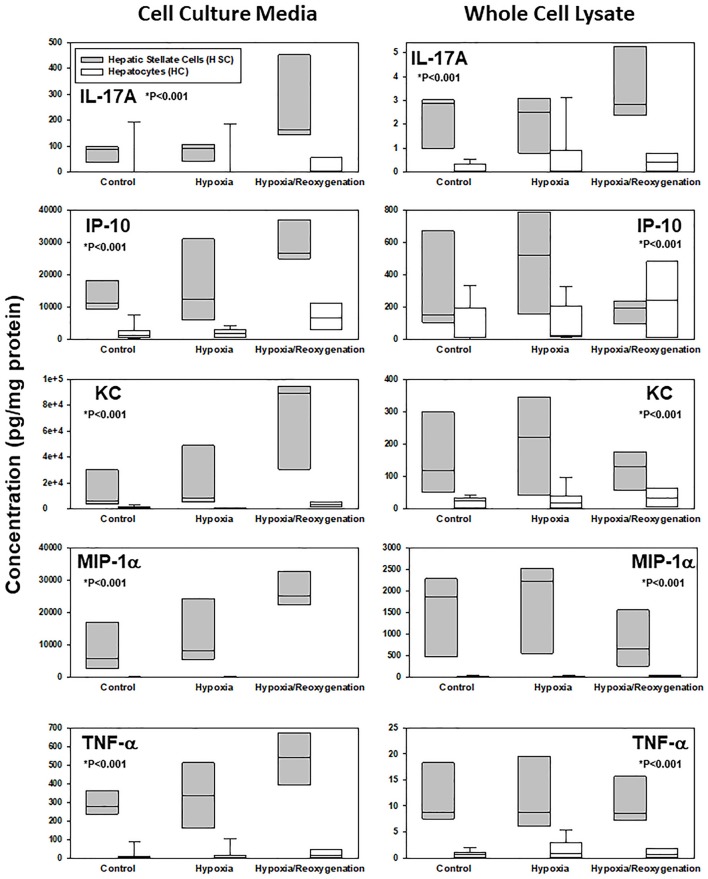
Differential response to hypoxia and hypoxia/reoxygenation in primary mouse hepatic stellate cells (HSC) and hepatocytes (HC). Freshly isolated HSC and HC from C57BL/6 mice were cultured under 21% O_2_ for 6 h (control), hypoxia (1% O_2_) for 6 h or hypoxia (6 h) followed by reoxygenation for 18 h. Inflammatory mediators were measured by Luminex™in both cell culture media and whole cell lysate as described in *Materials and Methods*. Box plots show the levels of IL-17A, IP-10, KC, MIP-1α, and TNF-α, where the line within the box marks the median and the lower and upper boundaries represent the 25th and 75th percentiles, respectively (^*^P < 0.001, HSC vs. HC, analyzed by Two-Way ANOVA).

**Figure 3:**
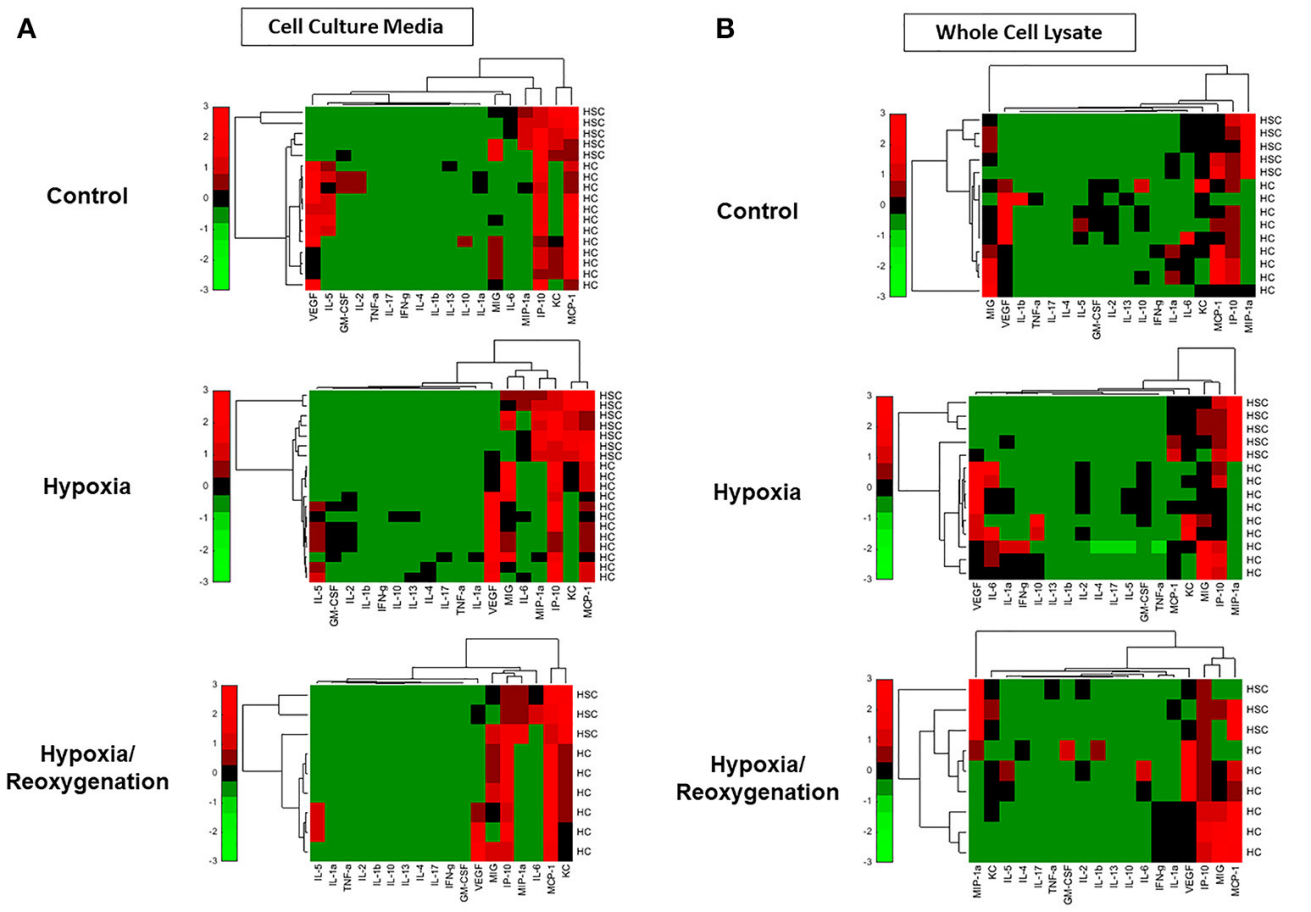
Hierarchical clustering of inflammatory mediators in mouse hepatic stellate cells (HSC) and hepatocytes (HC) and Freshly isolated HSC and HC from C57BL/6 mice were cultured under 21% O_2_ for 6 h (control), hypoxia (1% O_2_) for 6 h or hypoxia (6 h) followed by reoxygenation for 18 h. Inflammatory mediators were measured by Luminex™ in both cell culture media (CCM) **(A)** and whole cell lysate (WCL) **(B)** as described in *Materials and Methods*. Fold change values for each inflammatory mediator are represented in heat maps, ranging from large negative (green) to large positive values (red). No changes (zero values) are represented in black.

The production of inflammatory mediators in both CCM and WCL under the three experimental conditions, along with the corresponding *P*-values for **c**omparison of the changes in HSC vs. HC by Two-Way ANOVA, can be found in Supplementary Figure 1. Key examples of inflammatory mediators whose intracellular and extracellular levels are significantly different between HSC and HC are shown in Figure 2. In general, on a pg protein per mg total cell protein basis, HSC both produced and secreted much larger quantities of inflammatory mediators than the rather indolent response of HC. To identify those inflammatory mediators that showed similar expression or secretion behavior in each cell type, these patterns were then compared and grouped using hierarchical clustering. The resulting dendrograms showed a clear cluster separation between HC and HSC both in the CCM (Figure 3A) and WCL (Figure 3B) under the three experimental conditions studied. From this analysis, we infer that there is more heterogeneity in the intracellular (“thinking”) vs. the extracellular (“talking”) levels of inflammatory mediators in both cell types.

### Hypoxia and hypoxia/reoxygenation induce a differential response in Hsc vs. HC inferred from principal component analysis (PCA)

**Figure 4:**
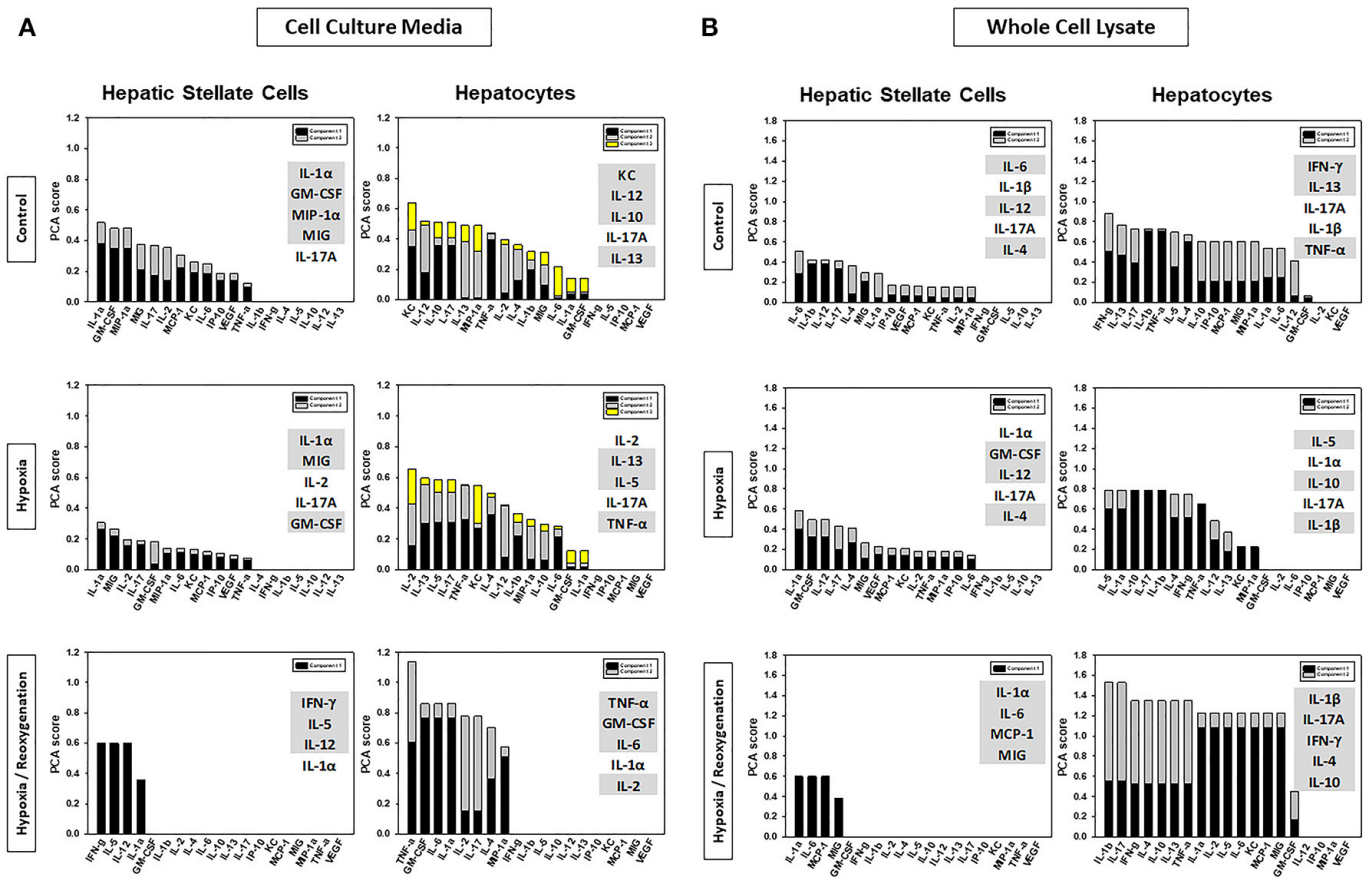
Comparison of HSC and HC based on Principal Component Analysis (PCA). Freshly isolated HSC and HC from C57BL/6 mice were cultured under 21% O_2_ for 6 h (control), hypoxia (1% O_2_) for 6 h or hypoxia (6 h) followed by reoxygenation for 18 h. Inflammatory mediators were measured by Luminex™ in both cell culture media (CCM) and whole cell lysate (WCL) and PCA was performed as described in *Materials and Methods*. Highlighted are the top five inflammatory mediators that contributed the most to the overall variance of the inflammatory response for each experimental condition in CCM **(A)** and WCL **(B)**.

Next, we sought to define an intermediate, core set of inflammation-associated characteristics of HSC and HC in response to increasing levels of stress, which we hypothesize represent different inflammation-related functions that ultimately become manifest in the expression and secretion of the defined subset of inflammatory mediators studied. To do so, we utilized PCA (Figure 1), a technique (or variants thereof) used by multiple investigators to delineate the core characteristics of a multivariate biological response (Janes et al., 2005; Janes and Yaffe, 2006; Mi et al., 2011; Nieman et al., 2012; Azhar et al., 2013; Ziraldo et al., 2013). We assessed several properties of the inflammatory responses under control, hypoxia, and hypoxia/reoxygenation: (1) the total number of mediators contributing to the total variance (i.e., mediators with sums of eigenvalues > 0; (2) the number of inferred principal components (represented as stacked bars), which we hypothesize represent distinct characteristics or inflammation-related functions; and (3) the sums of the eigenvalues (amplitude) for each experimental condition, which we hypothesize represents the magnitude of the inflammatory response to the indicated level of stress. The number of mediators and number of principal components expressed by each cell type under each condition is shown in Figure 4. An overall comparison of the inflammatory mediators showed that the inflammatory response of HSC was clearly different from that in HC both in CCM (Figure 4A) and WCL (Figure 4B) under each experimental condition. But certain similarities were seen. The major similarity was that the amplitude of the response was greatest when both HSC and HC were subjected to hypoxia/reoxygenation. Moreover, in general, the number of mediators involved as well as the inferred functions (number of principal components) in both WCL (“thinking”) and CCM (“talking”) were lowest in both HSC and HC subjected to hypoxia/reoxygenation as compared to control or hypoxia. A notable exception was the “thinking” response in HC WCL (Figure 4B), in which all three experimental conditions were characterized by two principal components, and the overall number of mediators was least in the hypoxia setting. Together, these analyses suggest that above a certain threshold of cellular stress, the inflammatory response generally becomes focused on a limited number of functions and associated mediators at a high intensity of response.

### Network analysis revealed the presence of distinct autocrine inflammatory networks in HSC vs. HC

The final analysis we carried out was aimed at discovering the initial networks of inflammation induced under control conditions, hypoxia, or hypoxia/reoxygenation (Figure 1). We have previously employed Dynamic Network Analysis (DyNA) to define the dynamic interconnections among inflammatory mediators in a mouse model of trauma/hemorrhagic shock (Mi et al., 2011), in isolated mouse HC subjected to hypoxic stress (Ziraldo et al., 2013), and to define the dynamics of systemic inflammation after trauma (Abboud et al., 2016; Namas et al., 2016) or during acute liver failure (Zamora et al., 2016). For the current analyses, we used a modified version of this method, in which data from a single experimental condition (rather than a time interval) were used.

**Figure 5:**
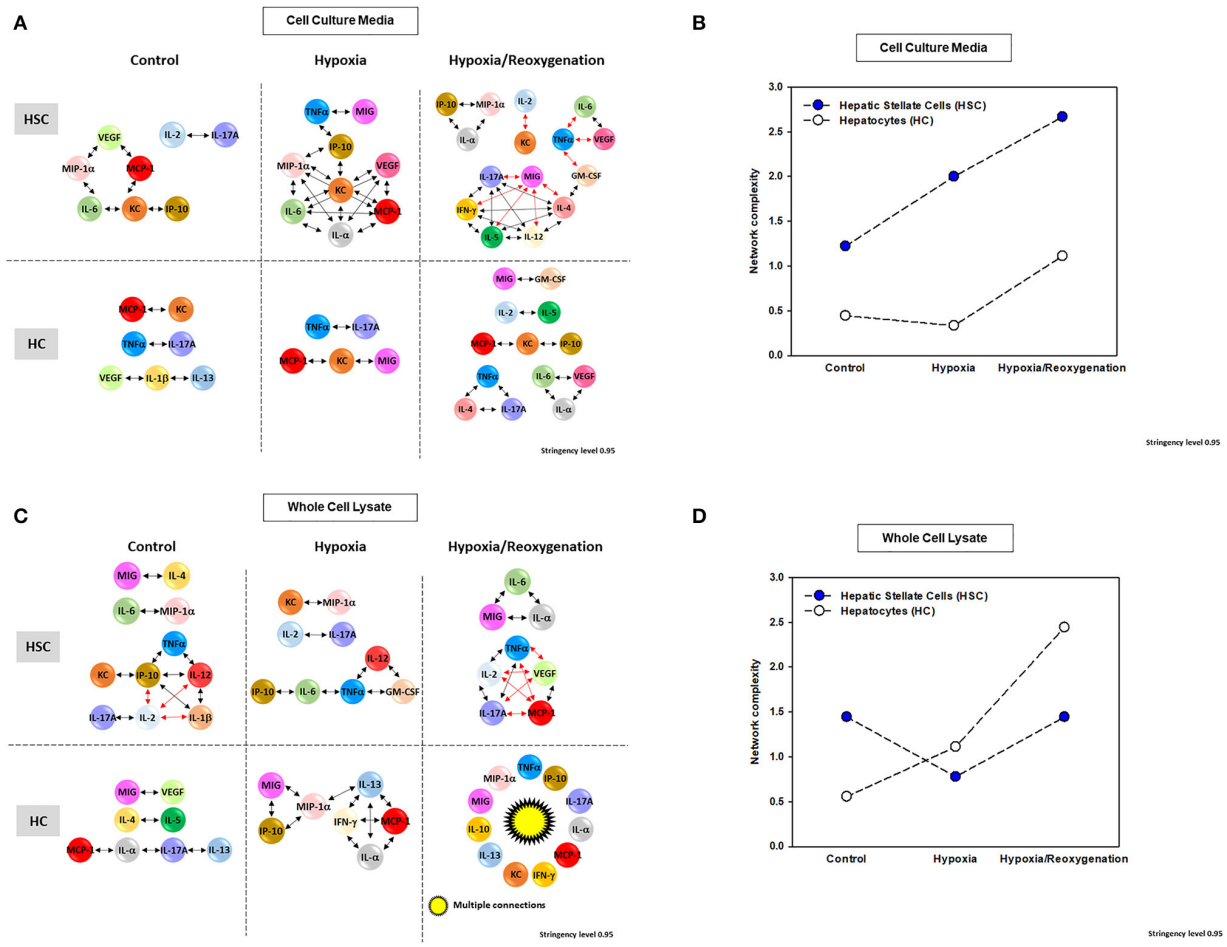
Network Analysis of inflammatory mediators in mouse hepatic stellate cells (HSC) and hepatocytes (HC) Freshly isolated HSC and HC from C57BL/6 mice were cultured under 21% O_2_ for 6 h (control), hypoxia (1% O_2_) for 6 h or hypoxia (6 h) followed by reoxygenation for 18 h. Inflammatory mediators released into cell culture media (CCM) and in whole cell lysate (WCL) were measured by Luminex™ and Network Analysis was performed as described in *Materials and Methods*. Figure shows the networks and the detailed connectivity (stringency level 0.95) for each experimental condition in both cell types in CCM **(A)** and WCL **(C)**. Black and red arrows represent positive and negative connections, respectively. **(B,D)** Show the network complexity in HSC vs. HC for CCM and WCL, respectively.

This network analysis suggested the presence of more complex extracellular inflammatory networks in HSC than in HC under all experimental conditions (Figures 5A,B and Supplementary Figure 2A) but roughly similarly complex intracellular networks (Figures 5C,D and Supplementary Figure 2B). Despite this difference in network complexity, multiple, shared inflammatory mediators were involved in both “thinking” and “talking” in HSC and HC. Secreted mediators shared by networks in both HSC and HC under control conditions included VEGF, MCP-1, KC, and IL-17A. Intracellularly under control conditions, only MIG and IL-4 were shared by HSC and HC networks. Under hypoxia, intracellular HSC and HC networks shared the mediators TNF-α, KC, MIG, and MCP-1, while intracellular networks only shared the mediators MIP-1α and IP-10. Under hypoxia/reoxygenation, secreted HSC and HC networks shared the greatest number of mediators: IL-1α, IL-2, IL-4, IL-6, IL-17A, MIG, KC, IP-10, GM-CSF, VEGF, and TNF-α; in contrast, shared intracellular mediators included TNF-α, MIG, MCP-1, IL-1α, and IL-17A. Control experiments comparing single network analysis of both isolated HC and HSC cultured under 21% O_2_ for 24 h confirmed the presence of more complex extracellular inflammatory networks in HSC than in HC under both control and hypoxia/reoxygenation (Supplementary Figure 3A) and similar complex intracellular networks after hypoxia/reoxygenation (Supplementary Figure 3B).

Based on PCA (Figure 4), we hypothesized that network interactions would lead to a reduced set of principal mediators and inflammatory functions in both CCM and WCL of HSC as they transition from hypoxia to hypoxia + reoxygenation. In support of this hypothesis, the networks observed under hypoxia/reoxygenation in HSC CCM as well as WCL exhibited multiple anti-correlated nodes (indicated in red; Figures 5A,C). We interpret this finding to mean that both intracellular and extracellular HSC mediators induced in the setting of hypoxia followed by reoxygenation regulate each other in a negative fashion that leads ultimately to the evolution of a reduced number of inflammatory functions relative to hypoxia alone. In the extracellular milieu of HSC, the chemokine MIG; the cytokines TNF-α, IL-6, and IL-17A; and the hypoxia-inducible growth factor VEGF appear to coordinate some of these downregulatory responses (Figure 5A). VEGF, TNF-α, MCP-1, and IL-17A also appear to play a more limited negative intracellular role (Figure 5B).

**Figure 6:**
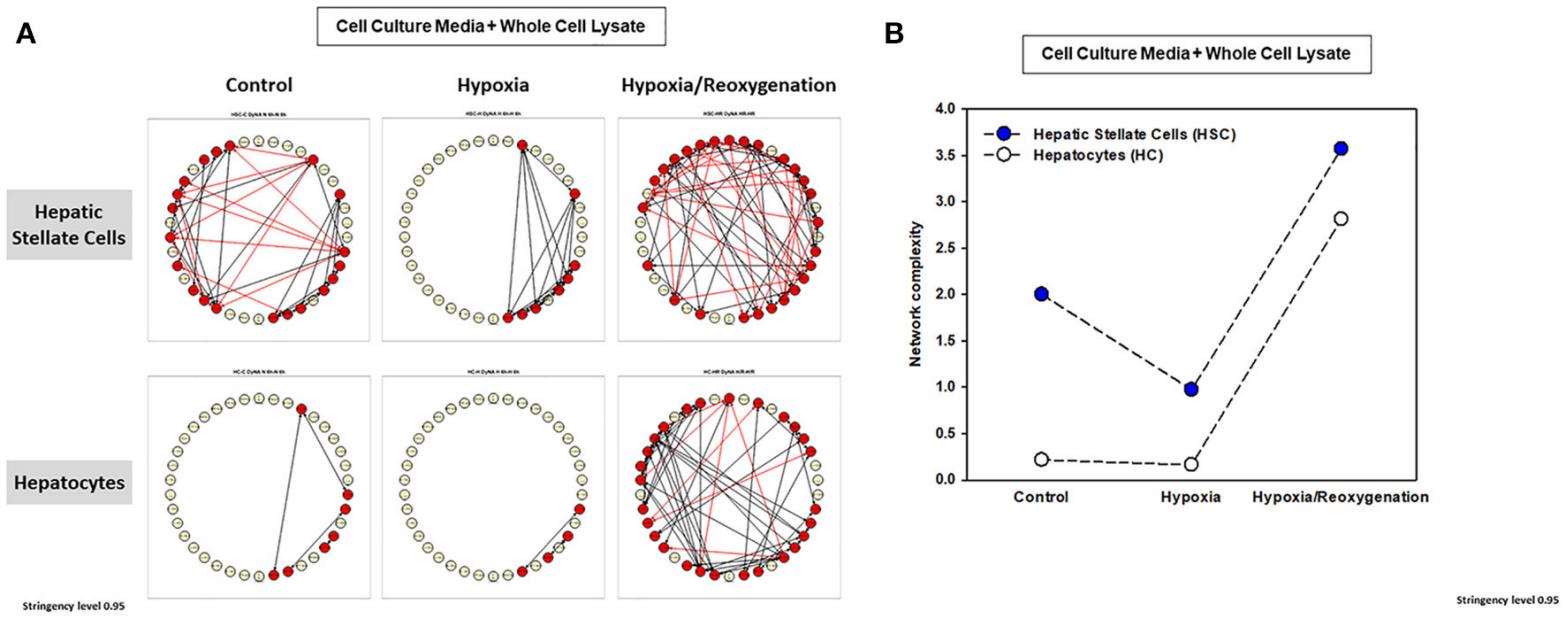
Network Analysis of inflammatory mediators using the combined data of both the cell culture media (CCM) and whole cell lysate (WCL) in mouse hepatic stellate cells (HSC) and hepatocytes (HC). Freshly isolated HSC and HC from C57BL/6 mice were cultured under 21% O_2_ for 6 h (control), hypoxia (1% O_2_) for 6 h or hypoxia (6 h) followed by reoxygenation for 18 h. Inflammatory mediators released into cell culture media (CCM) and in whole cell lysate (WCL) were measured by Luminex™ and Network Analysis was performed as described in *Materials and Methods*. The figure shows the networks and the detailed connectivity (stringency level 0.95) for each experimental condition combining the data in CCM and WCL **(A)**. Black and red arrows represent positive and negative connections, respectively. **(B)** Compares the combined network complexity in HSC vs. HC.

The foregoing analyses suggested that there may be an interplay between intracellular responses (“thinking”) and extracellular responses (“talking”) in each cell type. We therefore next sought to use network analysis to gain insights into the autocrine information flow within each cell population. Accordingly, we repeated the network analysis, but this time using both CCM and WCL data together for HSC vs. HC (Figure 6). Surprisingly, this analysis suggested the presence of multiple negative interactions in control HSC. This analysis also inferred the presence of a large number of negative interactions in both HSC and HC exposed to hypoxia/reoxygenation (Figure 6A,Table 1). Network analysis also demonstrated a higher complexity of autocrine networks involving both intracellular (“thinking”) and extracellular (“talking”) in HSC as compared to HC (Figure 6B), similar to the results obtained separately in CCM and WCL (Figures 5B,D).

**Table 1:** Total number of connections from Network Analysis.

**Network Analysis**	**Hepatic Stellate Cells (HSC)**		**Hepatocytes (HC)**	
	**Positive**	**Negative**	**Positive**	**Negative**
Control	24	13	4	0
Hypoxia	18	0	3	0
Hypoxia/Reoxygenation	39	27	45	7

*Freshly isolated hepatic stellate cells (HSC) and hepatocytes (HC) from C57BL/6 mice were cultured under 21% O_2_ for 6 h (control), hypoxia (1% O_2_) for 6 h or hypoxia (6 h) followed by reoxygenation for 18 h. Inflammatory mediators released into cell culture media (CCM) and in whole cell lysate (WCL) were measured by Luminex™and Network Analysis was performed as described in Materials and Methods (see Results in Figure 6A). Table shows the total number of positive (black arrows, see Figure 6A) and negative connections (red arrows, see Figure 6A) for each experimental condition in both cell types in CCM + WCL (stringency level 0.95)*.

We next assessed the flow of information from the intracellular (WCL) to extracellular milieu (CCM) in HSC vs. HC in greater detail (Figure 6 and Supplementary Table 1) Under control conditions, inflammatory networks in HC were comprised solely of extracellular mediators (IL-1β, IL-13, IL-17A, MCP-1, KC, VEGF, and TNF-α). In contrast, the combined network analysis inferred multiple positive and negative connections among a large number of intracellular and extracellular inflammatory mediators in HSC, suggesting a much more robust “thinking” and “talking” interplay in this cell type at baseline. Under hypoxia alone, both HSC and HC exhibited only positive, extracellular inflammatory connectivity, though the number of connections was higher in HSC (a large number of mediators) as compared to HC (only TNF-α, IL-17A, KC, MCP-1, and MIG). Interestingly, when subjected to hypoxia/reoxygenation, both HSC and HC exhibited a large number of positive and negative connections in the intracellular as well as extracellular compartments. This suggests a major adaptation to stress in HC, while this phenotype in HSC remained qualitatively similar to that observed under control conditions.

## Discussion

We report for the first time a detailed multiplex analysis of inflammatory mediators in isolated HSC and HC cultured under hypoxic conditions with and without reoxygenation, which suggests the presence of distinct, endogenous autocrine networks in isolated HSC and HC. The other cellular and structural components of the liver (vascular and sinusoidal endothelial cells, Kupffer cells, fibroblasts, biliary ducts, etc.) were excluded deliberately from these experiments in order to study the complexity of the autocrine responses of these two cellular components without the contribution of inflammatory mediators from contiguous cell types. Our data-driven computational work flow (see conceptual summary in Figure 1) was designed to elucidate how early inflammatory networks can lead to an intermediate set of responses that manifest ultimately in a generally restricted number of inflammatory mediators/functions that distinguish the responses of HSC from those of HC. Based on this work flow, we show that both cell types respond to hypoxic stress by releasing a somewhat similar, but not identical, repertoire of cytokines and chemokines *in vitro*. The similarities suggest the possible presence of a mutually supportive autocrine environment in the intact liver *in vivo*. The hypoxic stimulus evokes a much greater change in the secreted cellular inflammatory network of HSC than HC, indicating that the secretory machine in HC might be either more limited at baseline or disrupted following stress. Furthermore, the responses of HSC to hypoxia followed by reoxygenation are more vigorous and form more interactive networks than responses to hypoxia alone.

A significant role for HSC in regulating hepatic inflammatory and immunological responses by altering expression of numerous relevant genes has already been shown in LPS-stimulated rat HSC (Harvey et al., 2013). We have also shown previously that HSC produce numerous inflammatory mediators constitutively, expression of which is increased upon stimulation by LPS (Uemura and Gandhi, 2001; Thirunavukkarasu et al., 2005) (Thirunavukkarasu et al., 2008; Jameel et al., 2010; Harvey et al., 2013; Dangi et al., 2016). Furthermore, activation of unstimulated HSC stimulate DNA synthesis in HC (Uemura and Gandhi, 2001), and the mediators produced by LPS-stimulated HSC induce autophagy in HC as a survival mechanism but also cause apoptosis of a subset of cells (Dangi et al., 2016). We also reported a comprehensive analysis of LPS-induced expression of inflammatory mediators in HSC (Harvey et al., 2013). In the setting of hypoxia, we found that HSC-depleted mice are protected from ischemia/reperfusion injury, suggesting that under ischemic/hypoxic condition HSC release mediators that are injurious to HC (Stewart et al., 2014). Our analyses in the current study showed the presence of more complex autocrine inflammatory networks in HSC as compared to HC, which supports the notion that these two cell types not only coexist but also provide a microenvironment for each other in which HSC play the “messenger” role as opposed more of a “responder” role for HC. Experimental testing of this inference is part of ongoing and future experiments that will include other liver cells as well.

Our results suggest a complex interplay among chemokines (e.g., MCP-1, KC, MIG, and IP-10), cytokines (e.g., TNF-α and IL-17A, among many others), and growth factors (e.g., VEGF) in the response to hypoxia ± reoxygenation even in the absence of the contribution of other liver cells. Some of these cytokines have been reported to be induced by stress in HC and HSC previously (Kmieć, 2001; Brenner et al., 2013; Rani et al., 2017). Our computational analysis, however, elucidate a novel dimension of information regarding how HSC and HC “think” and “talk”: as stress increases, intracellular and extracellular information transfer appears to result in a reduction of inflammation-related functions, a process we hypothesize is driven through negative network interactions among key inflammatory mediators.

Finally, we are aware of the limitations in translating these computational analyses *in vivo*. Under physiological conditions O_2_ is a major effector of metabolic zonation and plays a major role in the liver. When compared to other somatic cells, liver cells are relatively hypoxic, both in the periportal (zone 1, 9–11% O_2_) and the perivenous region (zone 3, 5–7% O_2_) (Jungermann and Kietzmann, 2000). Changes in this complex O_2_ gradient, as well as in the production of hormones and enzymes, affect the response of the liver in a number of pathophysiological processes. Thus, we agree that virtually all routine tissue culture experiments involving liver cells, such as the control conditions described herein are normally performed under relatively hyperoxic conditions that may not reflect *in vivo* physiology (Sullivan et al., 2006). Notably, the complex O_2_ gradient in the liver is very difficult to duplicate using isolated cells *in vitro*. Furthermore, it is also difficult to identify (and quantify) the presence and activity of specific inflammatory or parenchymal cells during specific time periods, as is the identification of potential crosstalk among dynamic networks *in vivo*. Although it is very likely, whether or not these inflammatory networks change in both composition and complexity when HC and HSC are in close proximity, as it happens *in vivo*, has not yet been reported and it represents another interesting element in the complex relationship between liver cells. To elucidate this and other of our hypotheses will require a more detailed analysis of data obtained from co-cultures of HC with HSC both alone and in combination with other relevant cells in the liver such as Kupffer cells and endothelial cells. Future studies will be directed to address these limitations, specifically we have ongoing studies in primary mouse HC as well as in plasma and whole liver in a mouse model of ischemia/reperfusion.

In summary, our systems-based analysis suggests the presence of an inflammatory homeostatic environment in resting isolated liver cells, which is disrupted under stress such as hypoxia and hypoxia followed by reoxygenation. The vigorous production of secreted inflammatory mediators from HSC and the relative indolent response from isolated HC suggest that HSC act as resident transformers of external stimuli or blood-borne for other less responsive cells within the complex cellular structure of the liver. The relevance of these findings *in vivo* warrants further investigation.

## Author contributions

YV participated in computational and statistical analysis, data interpretation, and writing. RS participated in data interpretation and writing. CG participated in data interpretation and writing. DB participated in Luminex analysis. BJ participated in cell culture experiments (HC). Fe-D participated in data analysis. QM participated in statistical and computational analysis. CH participated in cell culture experiments (HSC). TB participated in data interpretation and writing. RZ participated in study design, computational and statistical analysis, data interpretation, and writing. RN participated in cell culture experiments (HSC).

### Conflict of interest statement

The authors declare that the research was conducted in the absence of any commercial or financial relationships that could be construed as a potential conflict of interest.

## Supplementary material

Supplementary Figure 1 Production of inflammatory mediators in mouse hepatic stellate cells (HSC) and hepatocytes (HC). Freshly isolated HSC and HC from C57BL/6 mice were cultured under 21% O_2_ for 6 h (control), hypoxia (1% O_2_) for 6 h or hypoxia (6 h) followed by reoxygenation for 18 h. Inflammatory mediators were measured by Luminex™ in both cell culture media and whole cell lysate as described in *Materials and Methods*. Results shown represent mean ± SEM (^*^*P* < 0.05, HSC vs. HC, analyzed by Two-Way ANOVA).

Supplementary Figure 2 Network Analysis of inflammatory mediators in mouse hepatic stellate cells (HSC) and hepatocytes (HC). Freshly isolated HSC and HC from C57BL/6 mice were cultured under 21% O_2_ for 6 h (control), hypoxia (1% O_2_) for 6 h or hypoxia (6 h) followed by reoxygenation for 18 h. Inflammatory mediators released into cell culture media (CCM) and in whole cell lysate (WCL) were measured by Luminex™ and Network Analysis was performed as described in *Materials and Methods*. Figure shows the networks (stringency level 0.95) for each experimental condition in both cell types in CCM **(A)** and WCL **(B)**. Black and red arrows represent positive and negative connections, respectively.

Supplementary Figure 3 Network Analysis of inflammatory mediators in mouse hepatic stellate cells (HSC) and hepatocytes (HC). Freshly isolated HSC and HC from C57BL/6 mice were cultured under 21% O_2_ for 24 h (control) or hypoxia (6 h) followed by reoxygenation for 18 h (as in Supplementary Table 1). Inflammatory mediators released into cell culture media (CCM) and in whole cell lysate (WCL) were measured by Luminex™ and Network Analysis was performed as described in *Materials and Methods*. Figure shows the networks (stringency level 0.95) for each experimental condition in both cell types in CCM **(A)** and WCL **(B)**. Black and red arrows represent positive and negative connections, respectively.

Supplementary Table 1 Network Analysis of inflammatory mediators using the combined data of both the cell culture media (CCM) and whole cell lysate (WCL) in mouse hepatic stellate cells (HSC) and hepatocytes (HC). Freshly isolated HSC and HC from C57BL/6 mice were cultured under 21% O_2_ for 6 h (control), hypoxia (1% O_2_) for 6 h or hypoxia (6 h) followed by reoxygenation for 18 h. Inflammatory mediators released into cell culture media (CCM) and in whole cell lysate (WCL) were measured by Luminex® and Network Analysis was performed as described in *Materials and Methods*. The figure shows the detailed connectivity (stringency level 0.95) for each experimental condition combining the data in CCM and WCL.

## Abbreviations

GM-CSF: Granulocyte-Macrophage Colony Stimulating Factor
IFN: Interferon
IL: interleukin
IP-10: IFN-γ-inducible Protein of 10 kDa/CXCL10
KC: Keratinocyte-derived Cytokine/CXCL1
MCP-1: Monocyte Chemotactic Protein-1/CCL2
MIG: Monokine induced by γ-interferon/CXCL9
MIP-1α: Macrophage Inflammatory Protein-1α (MIP-1α/CCL3α)
TNF-α: Tumor Necrosis Factor-α
VEGF: Vascular Endothelial Growth Factor
PCA: Principal Component Analysis.
