# Supplementary figures and images for: “Thinking” vs. “Talking”: Differential Autocrine Inflammatory Networks in Isolated Primary Hepatic Stellate Cells and Hepatocytes under Hypoxic Stress
Source: Front Physiol. 2017 Dec 22;8:1104. doi: 10.3389/fphys.2017.01104 (PMC5743931; doi:10.3389/fphys.2017.01104)

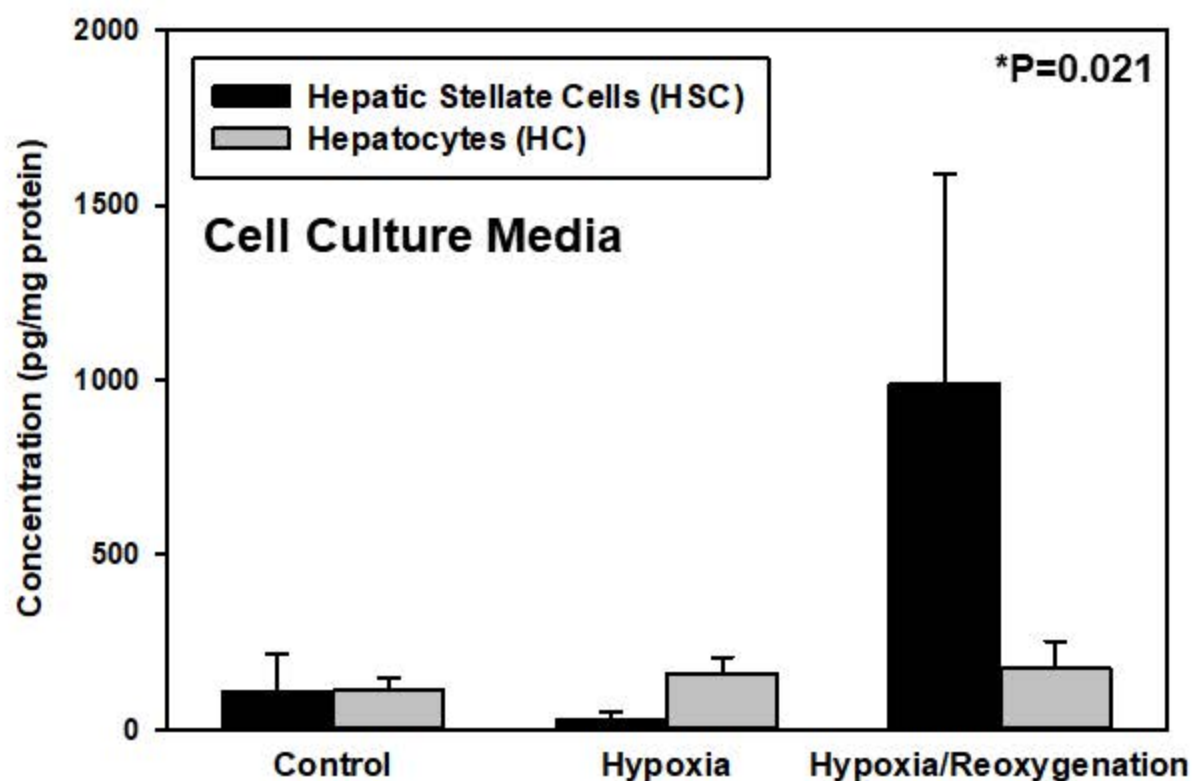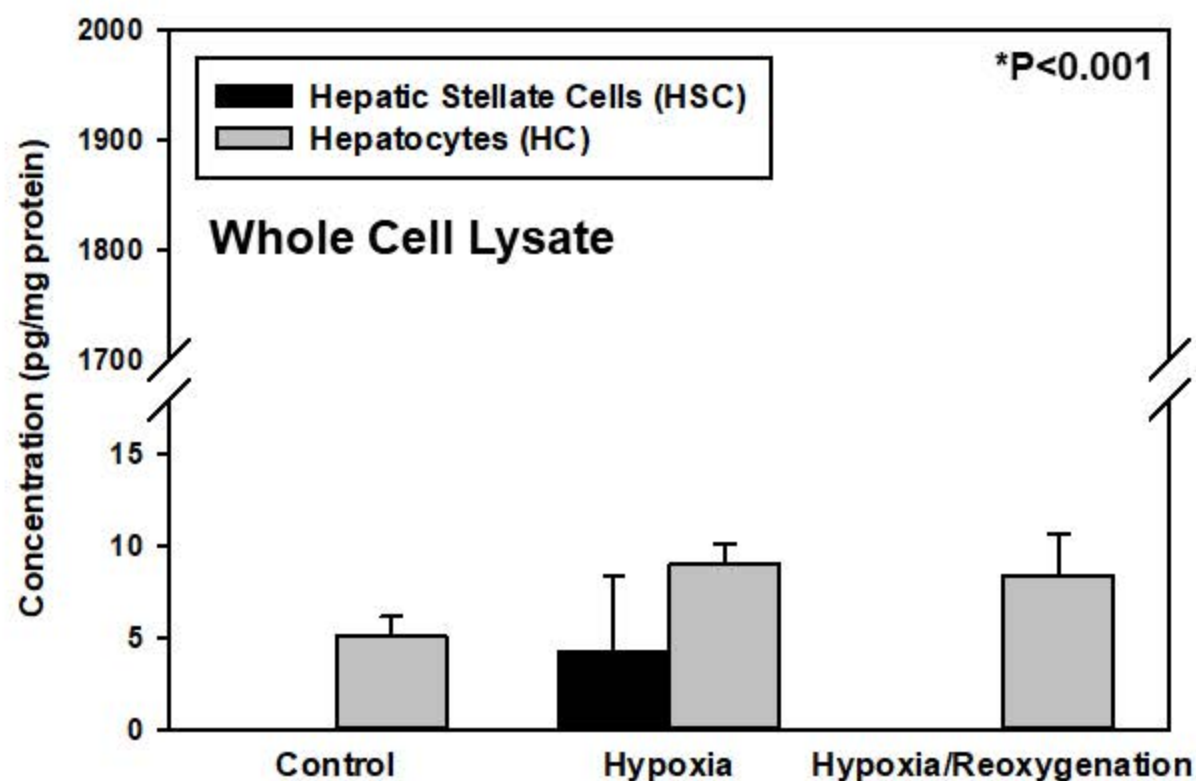

Suppl. Fig. 1

IFN- $\gamma$

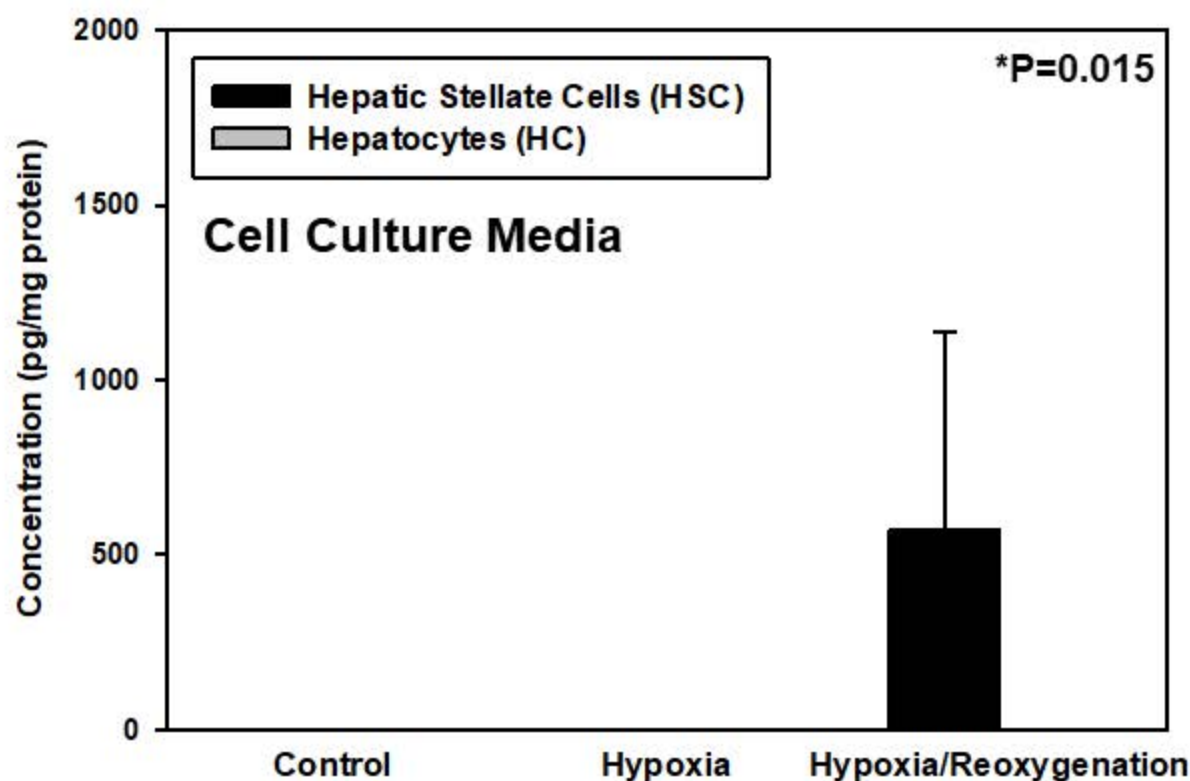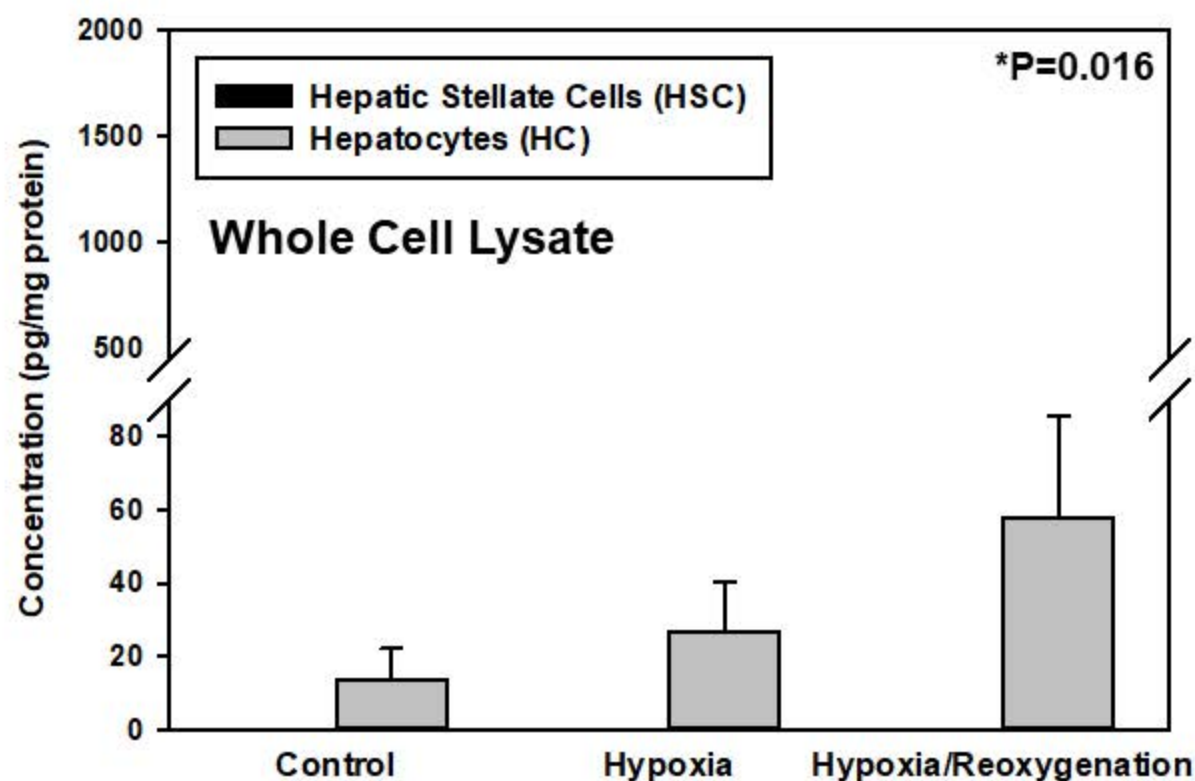

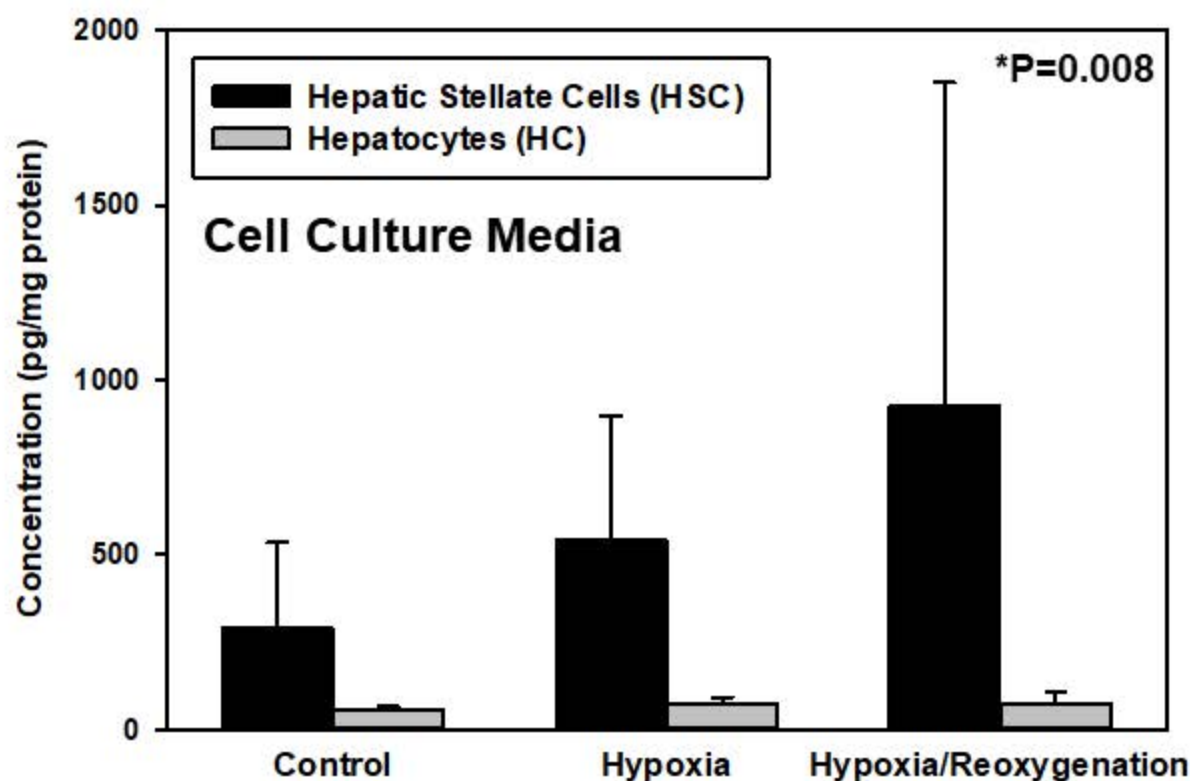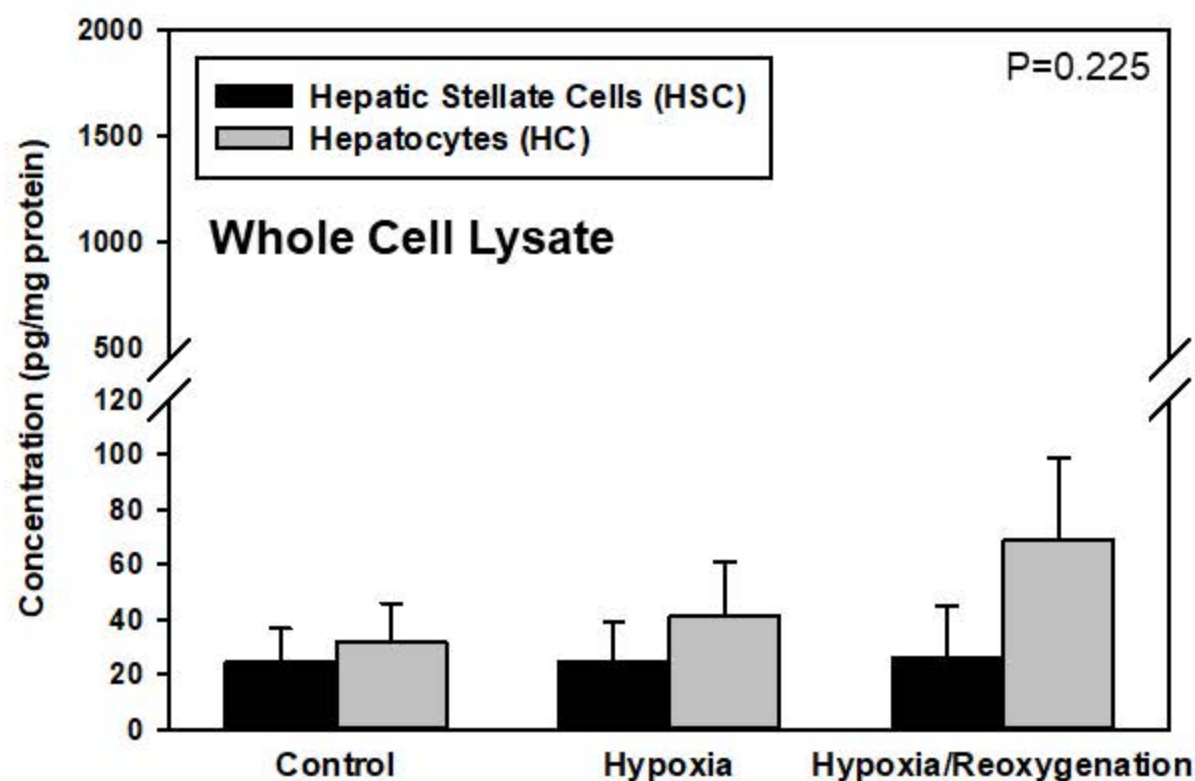

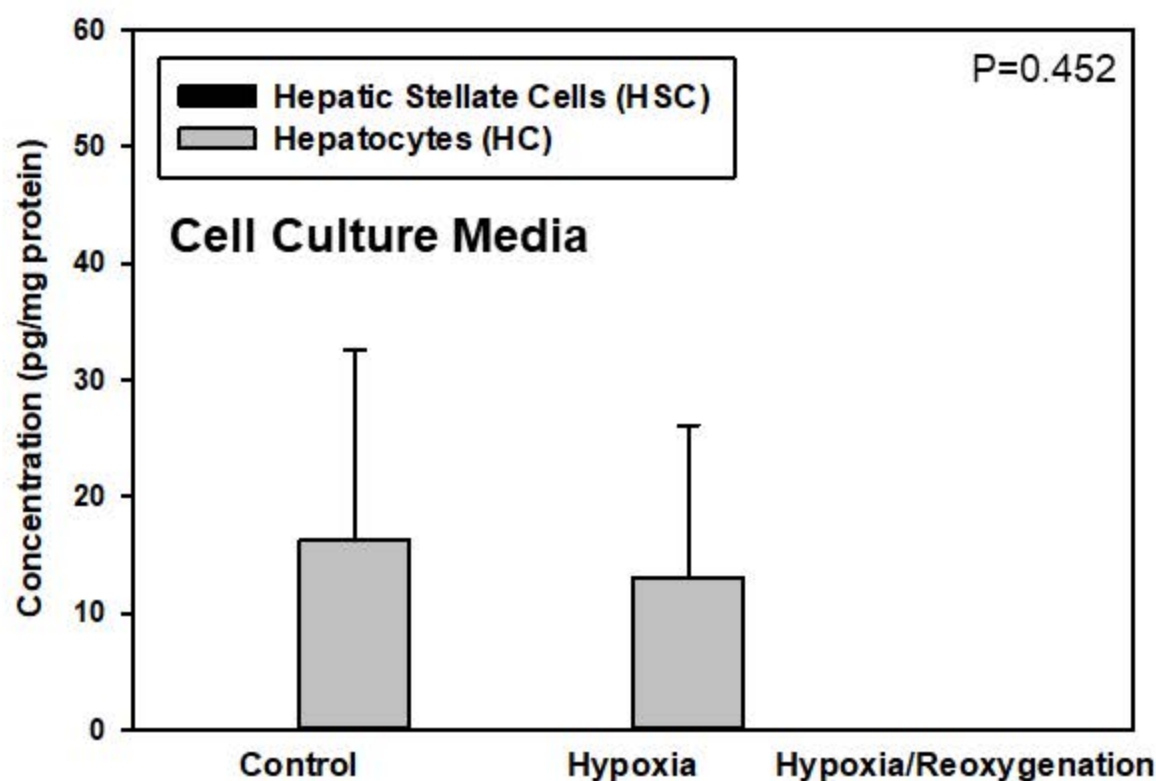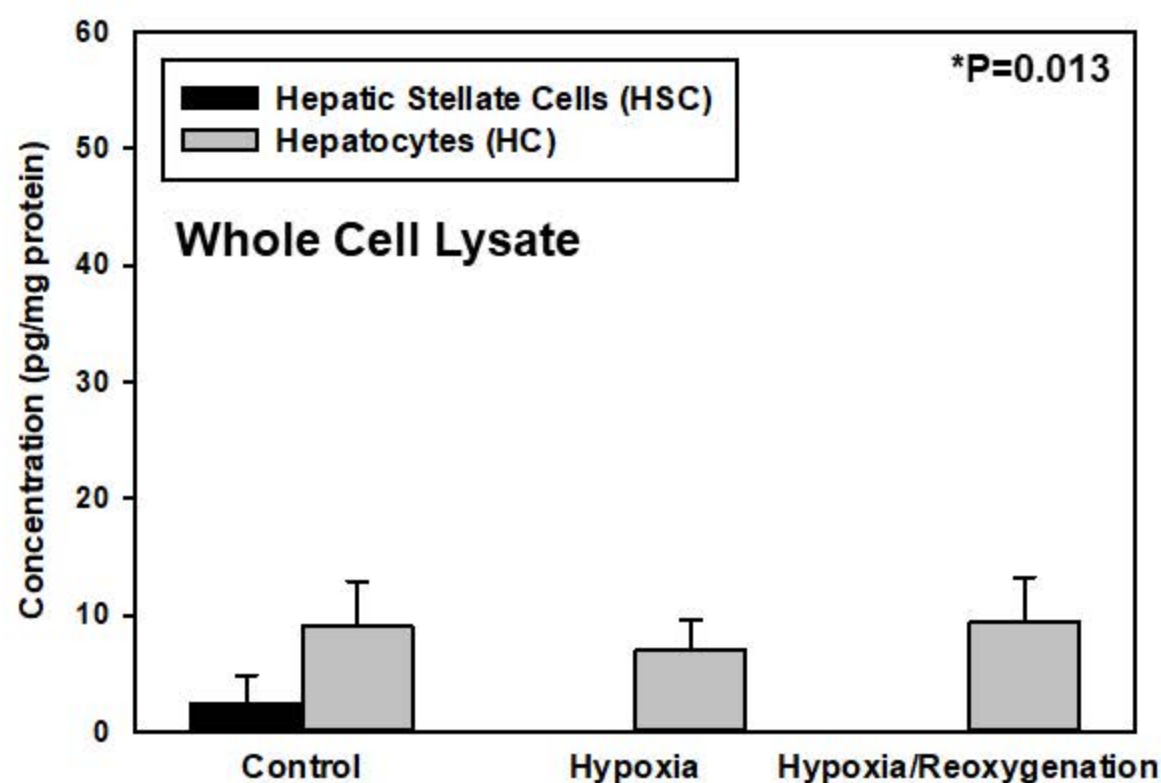

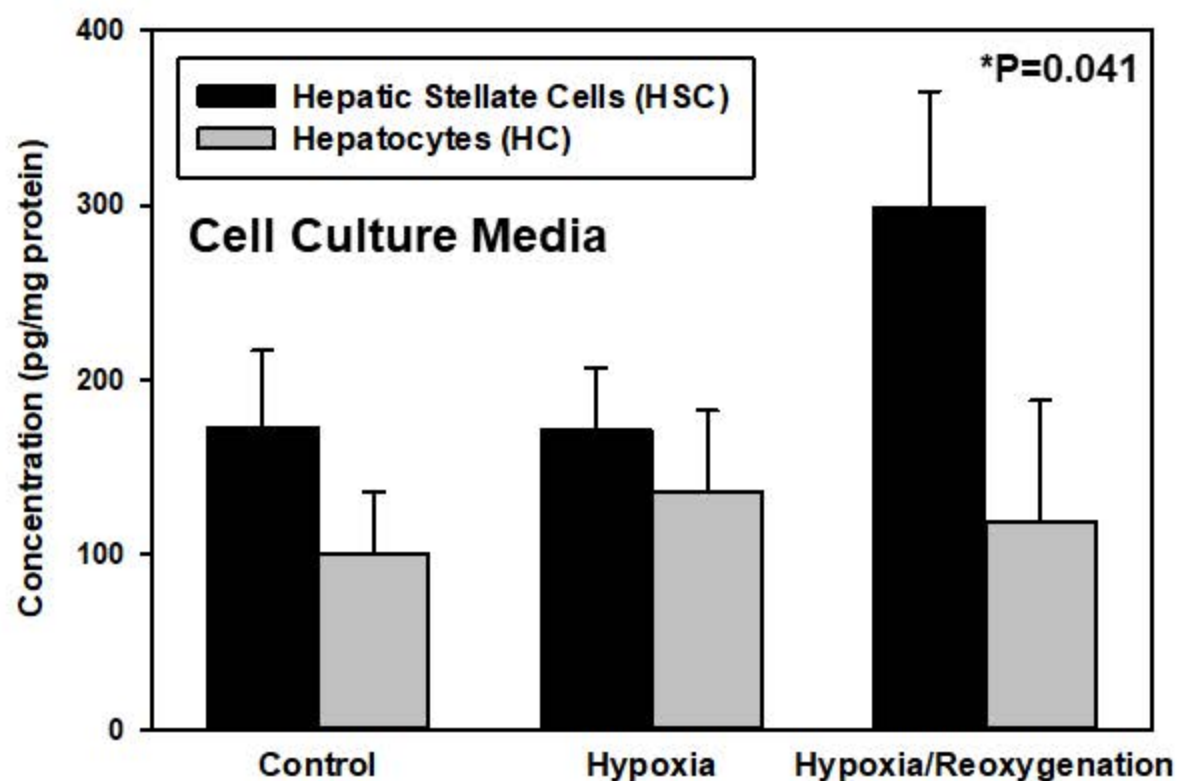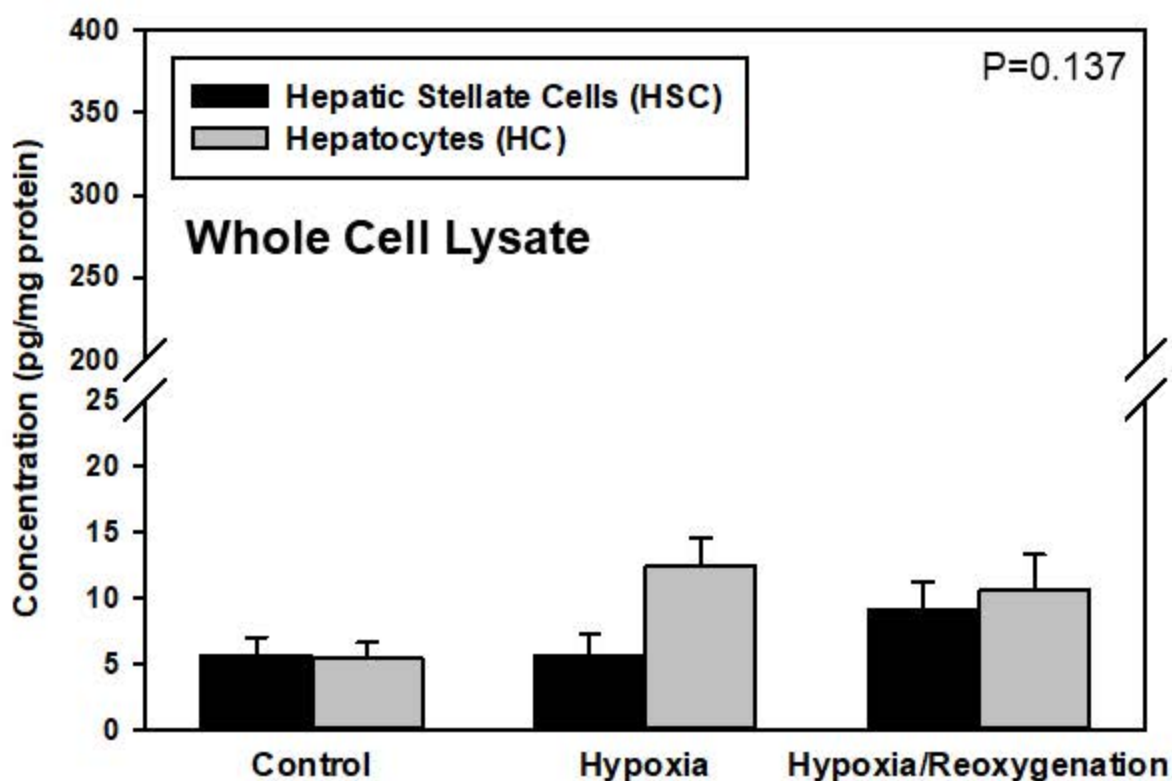

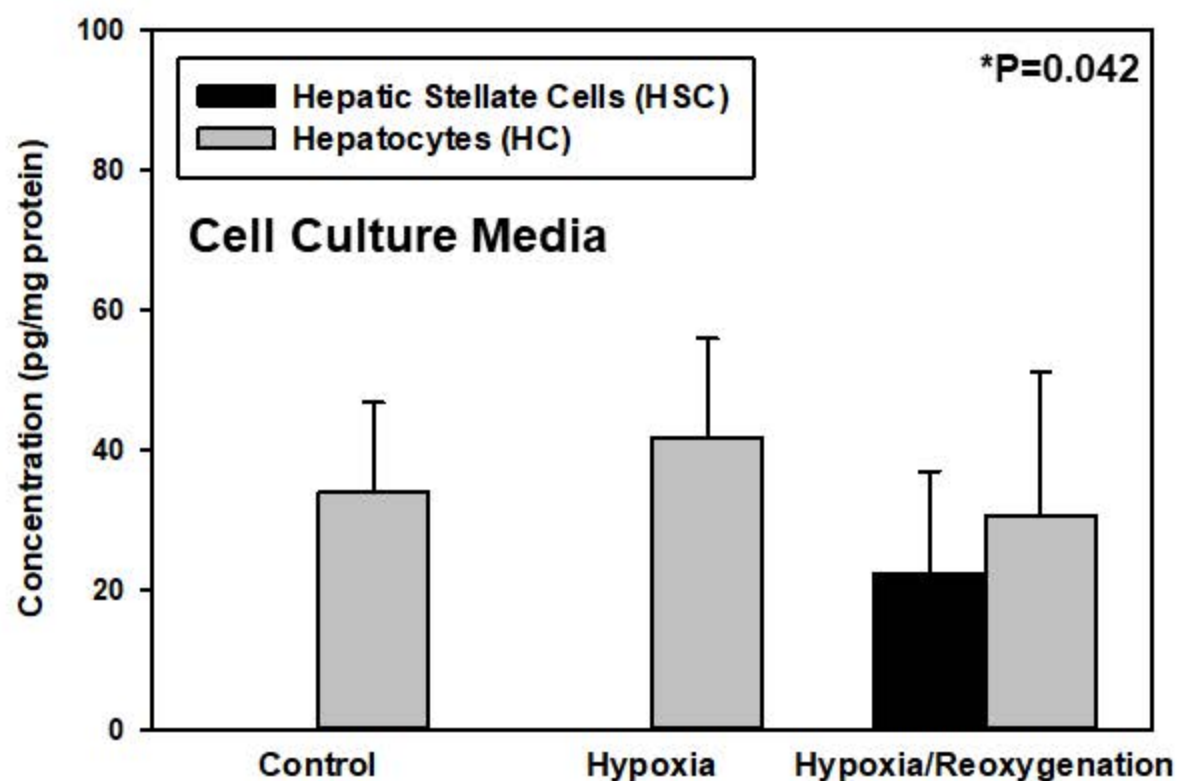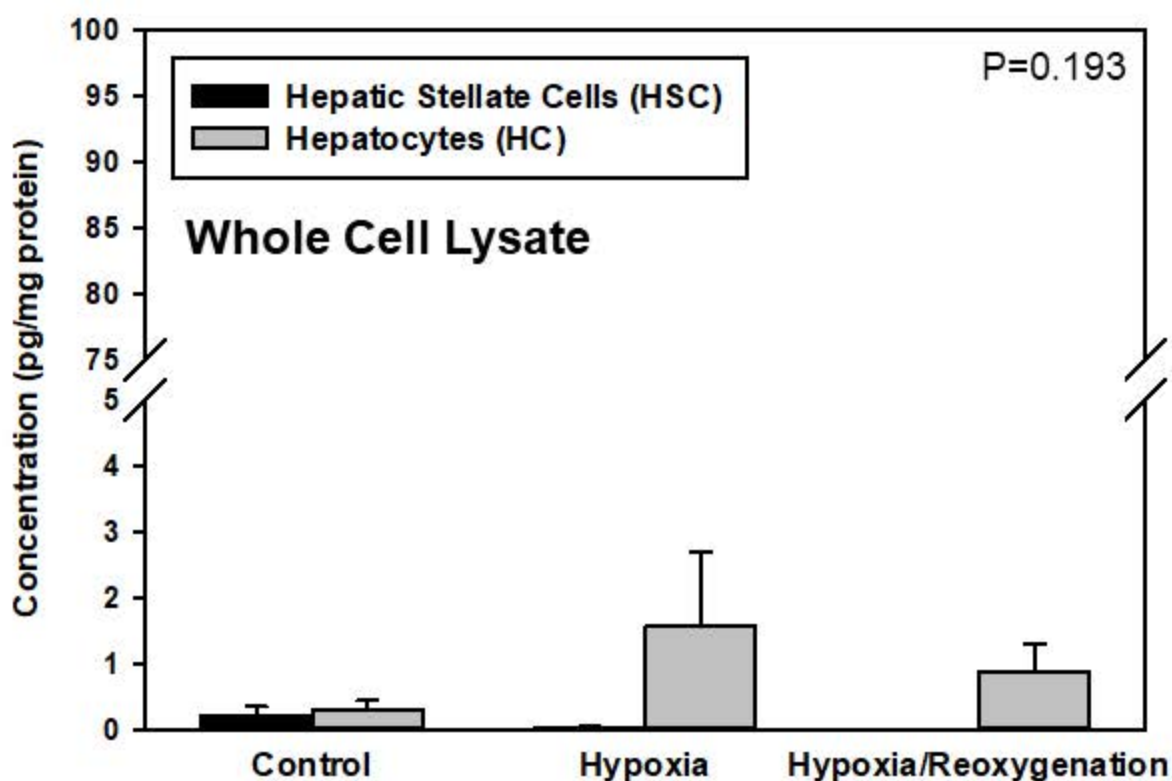

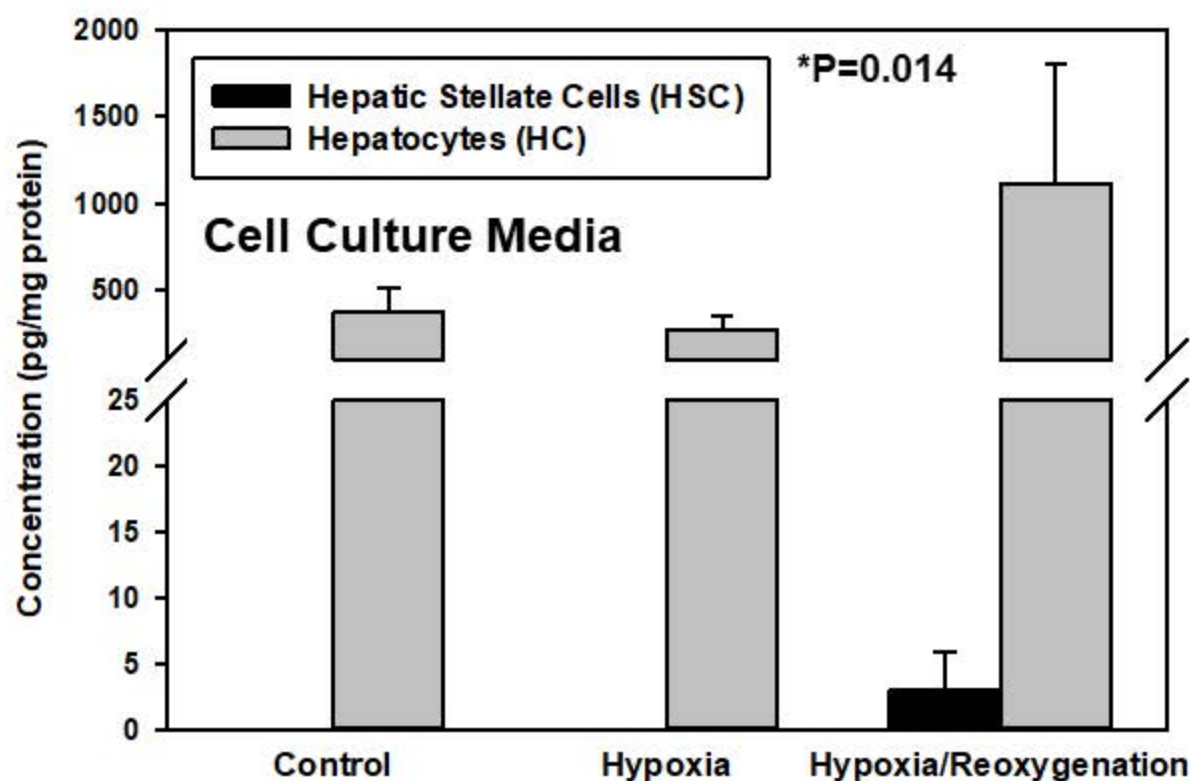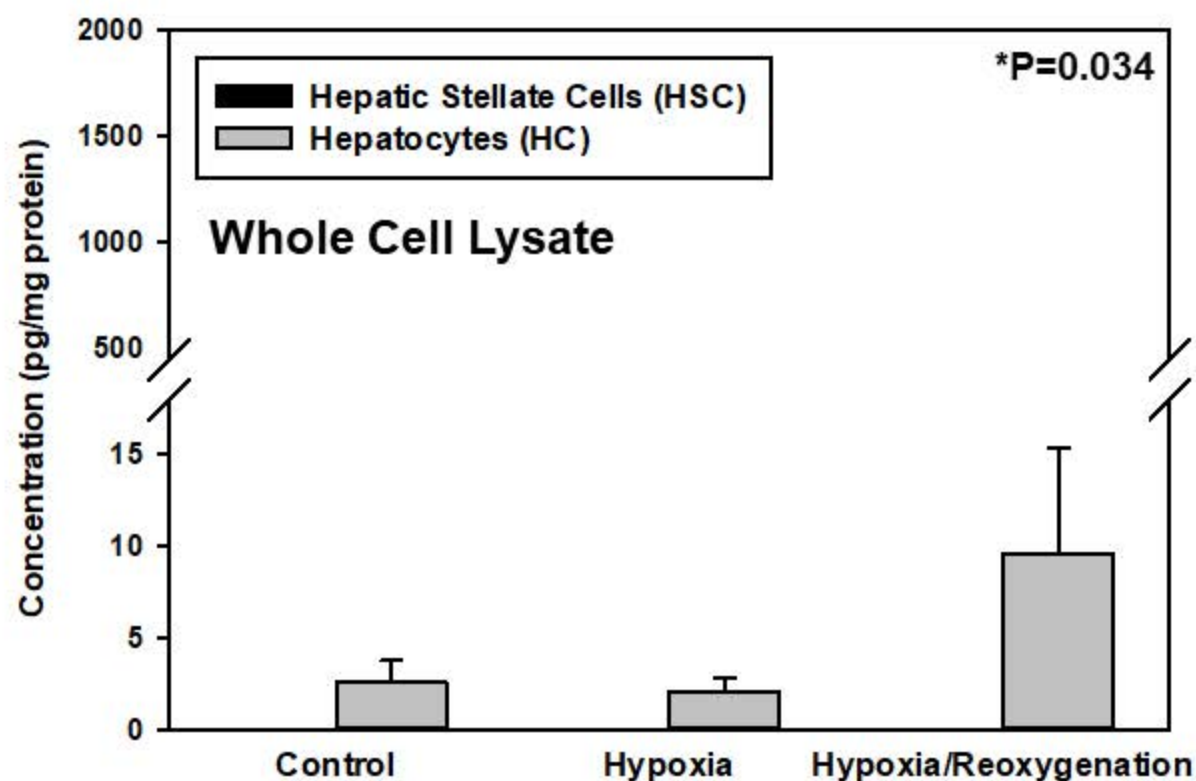

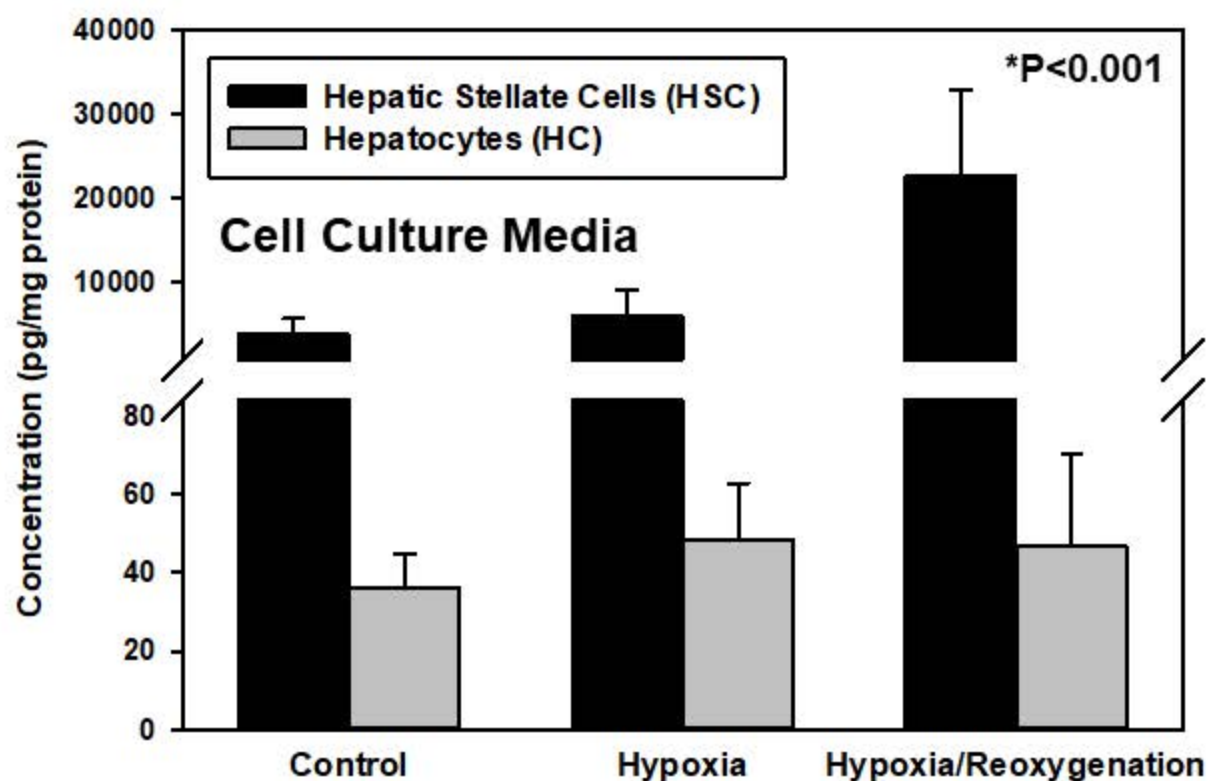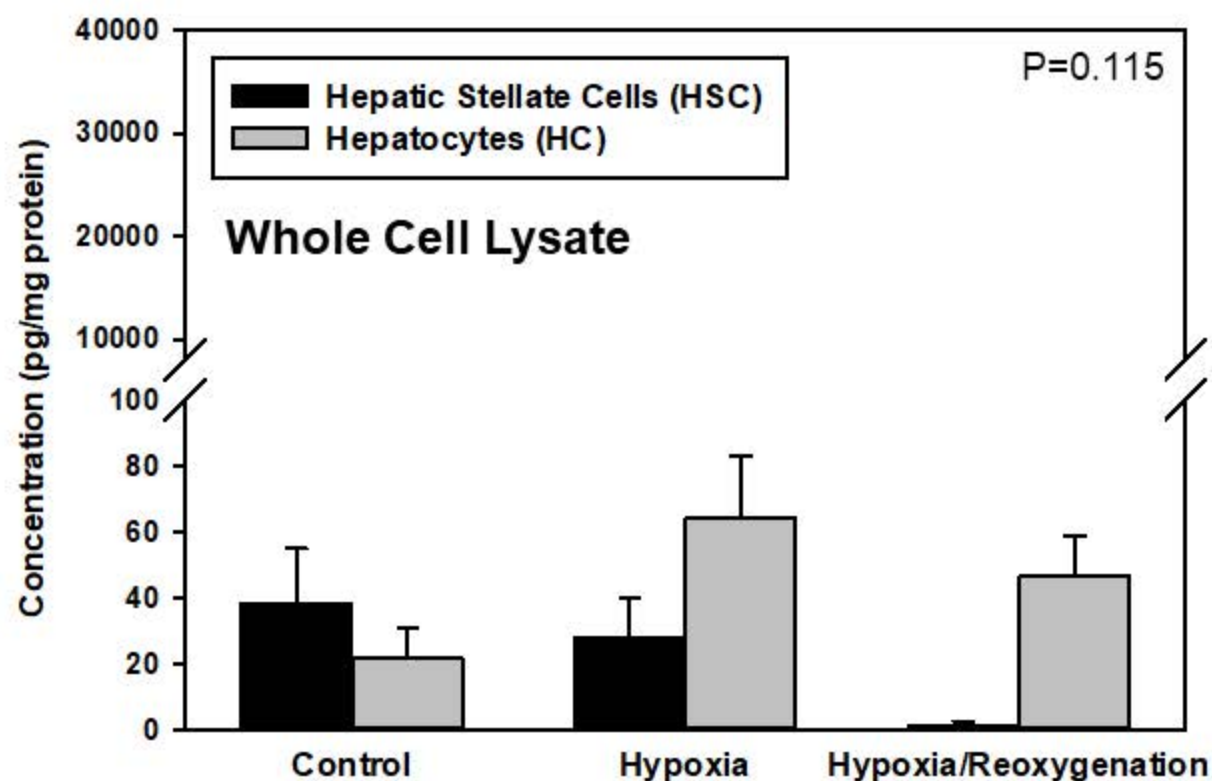

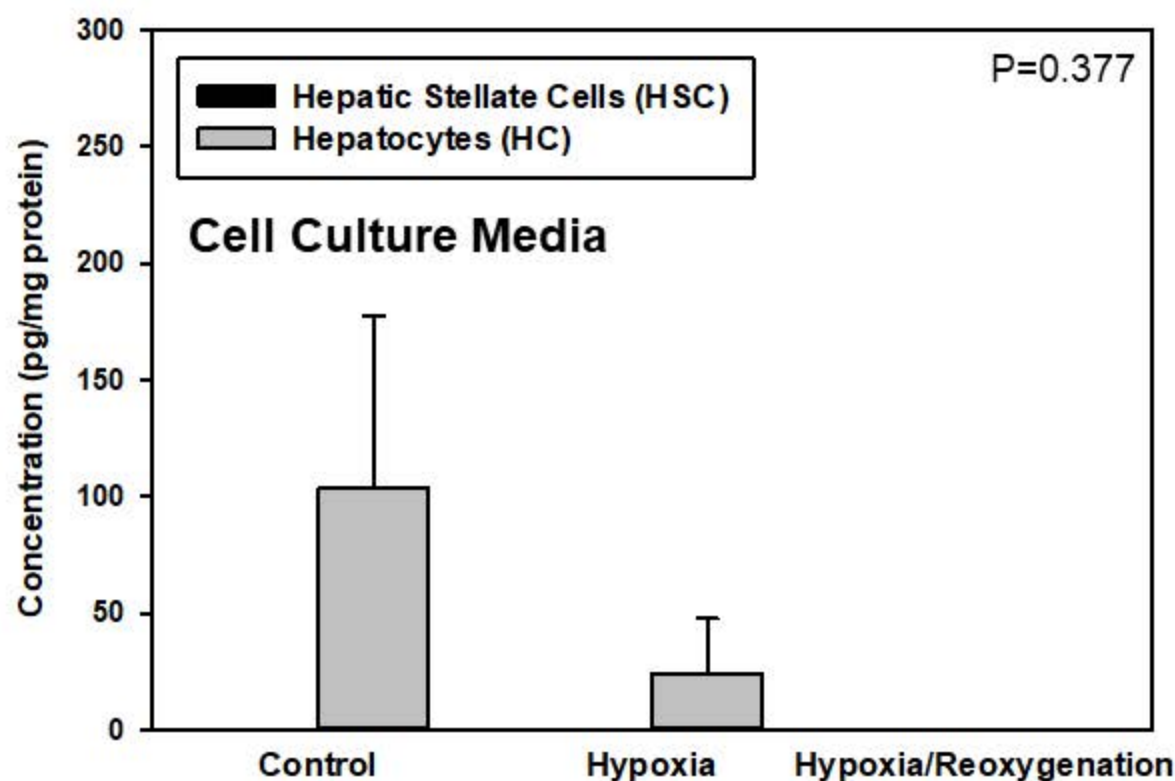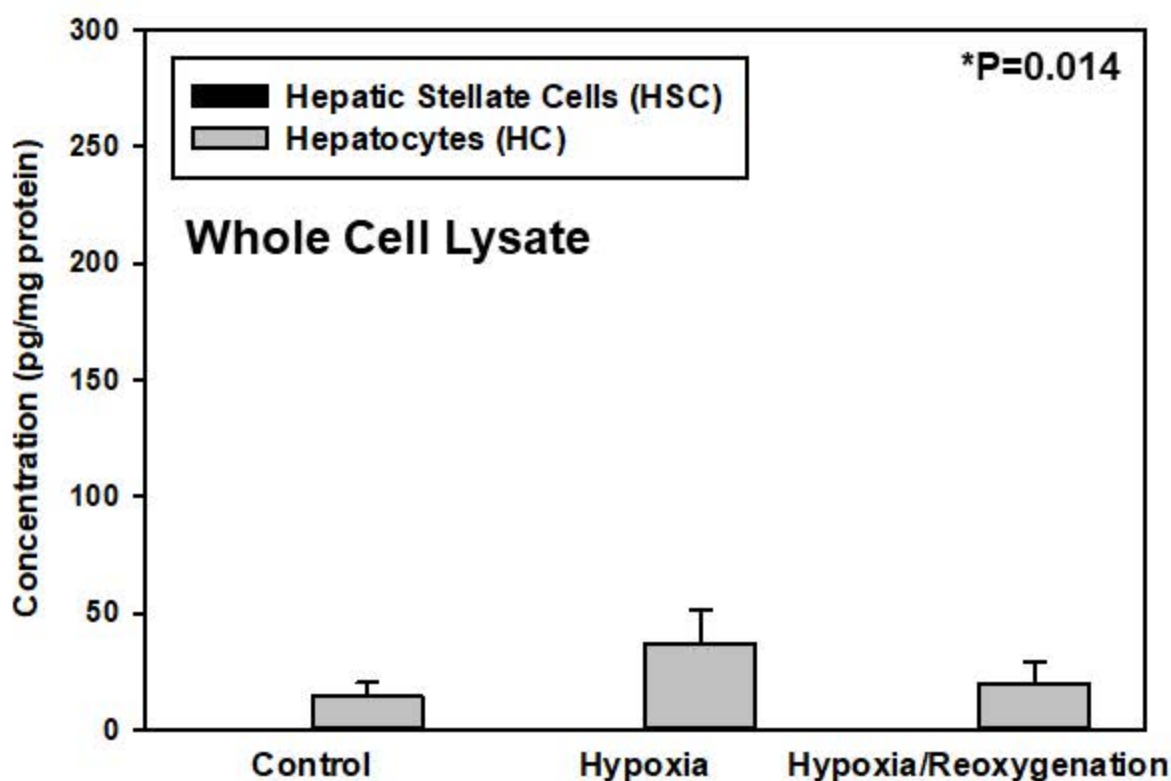

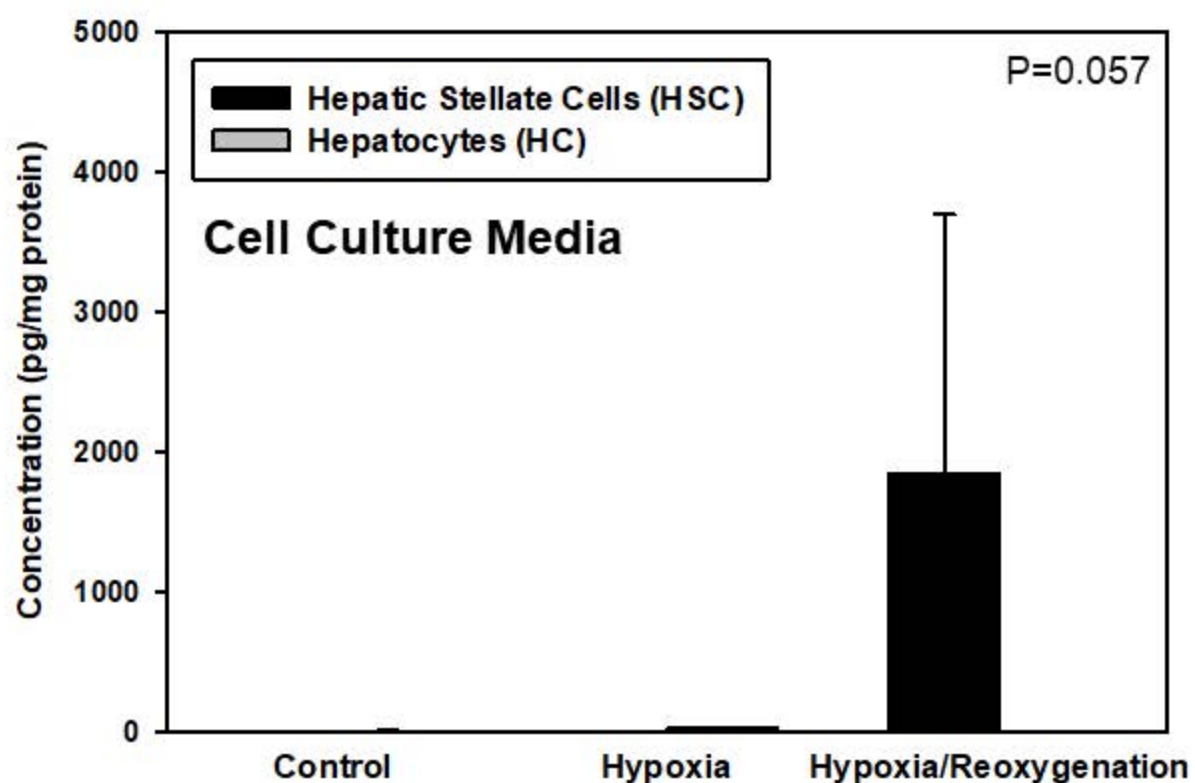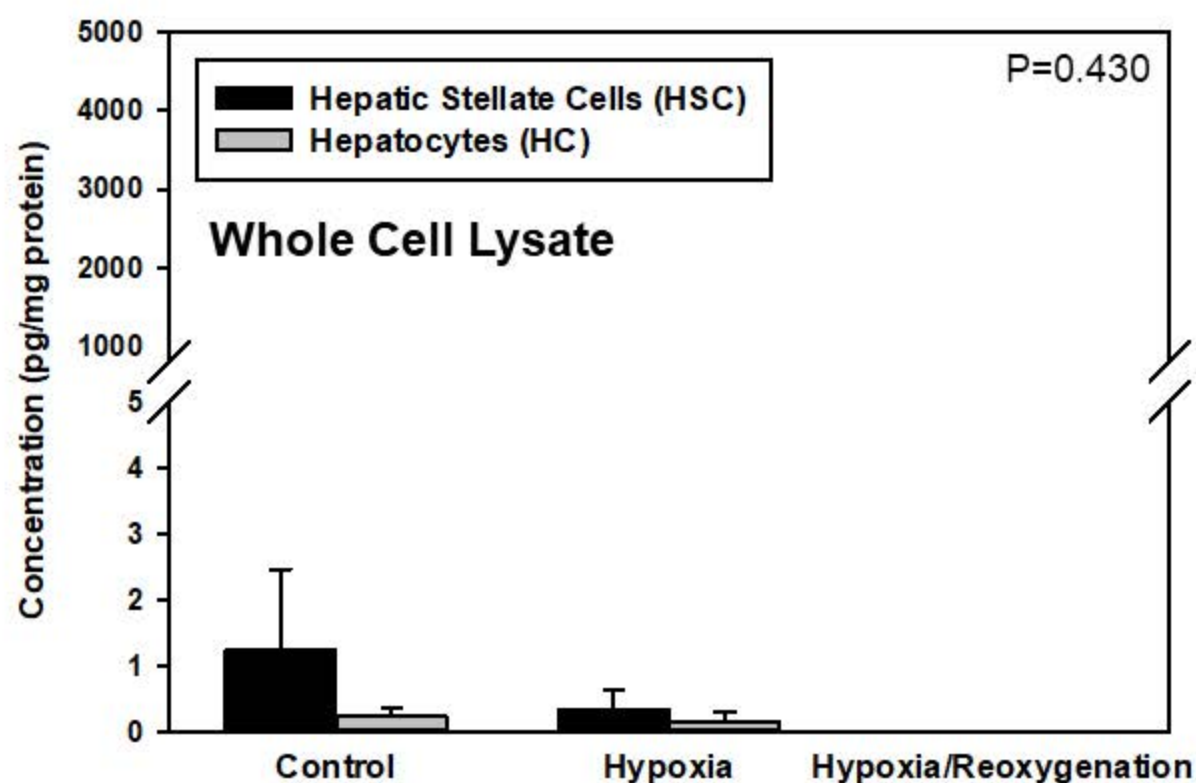

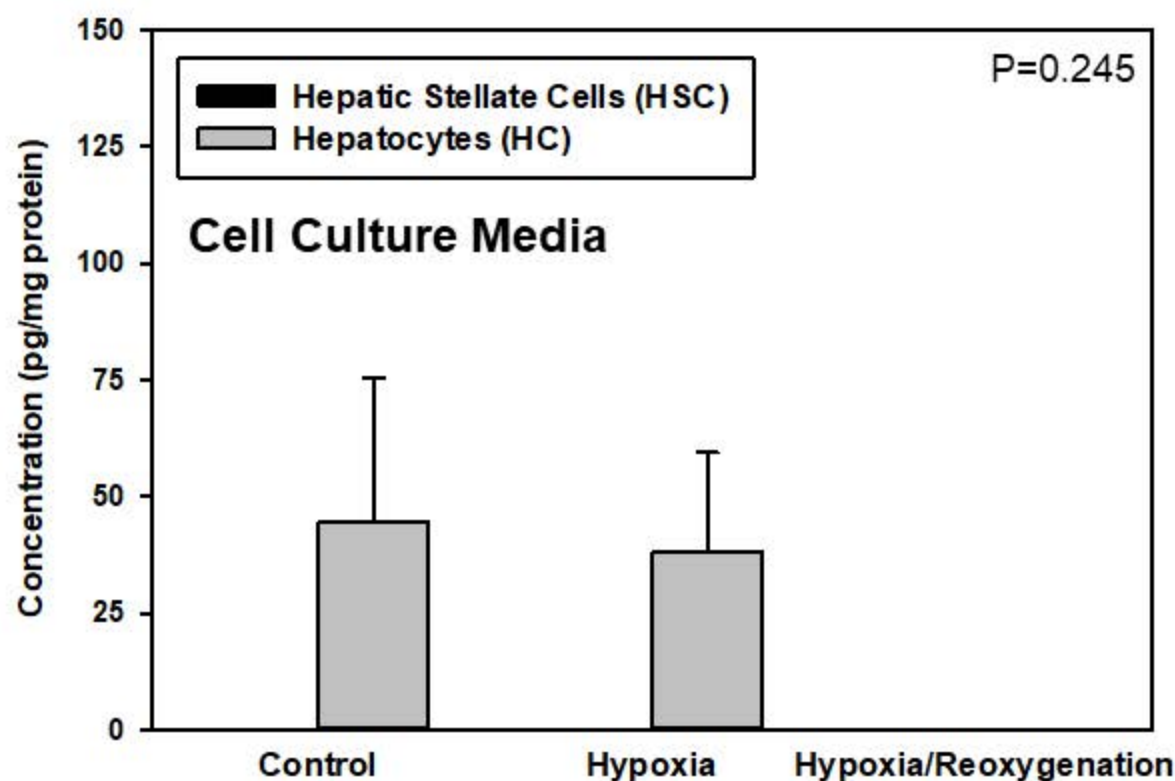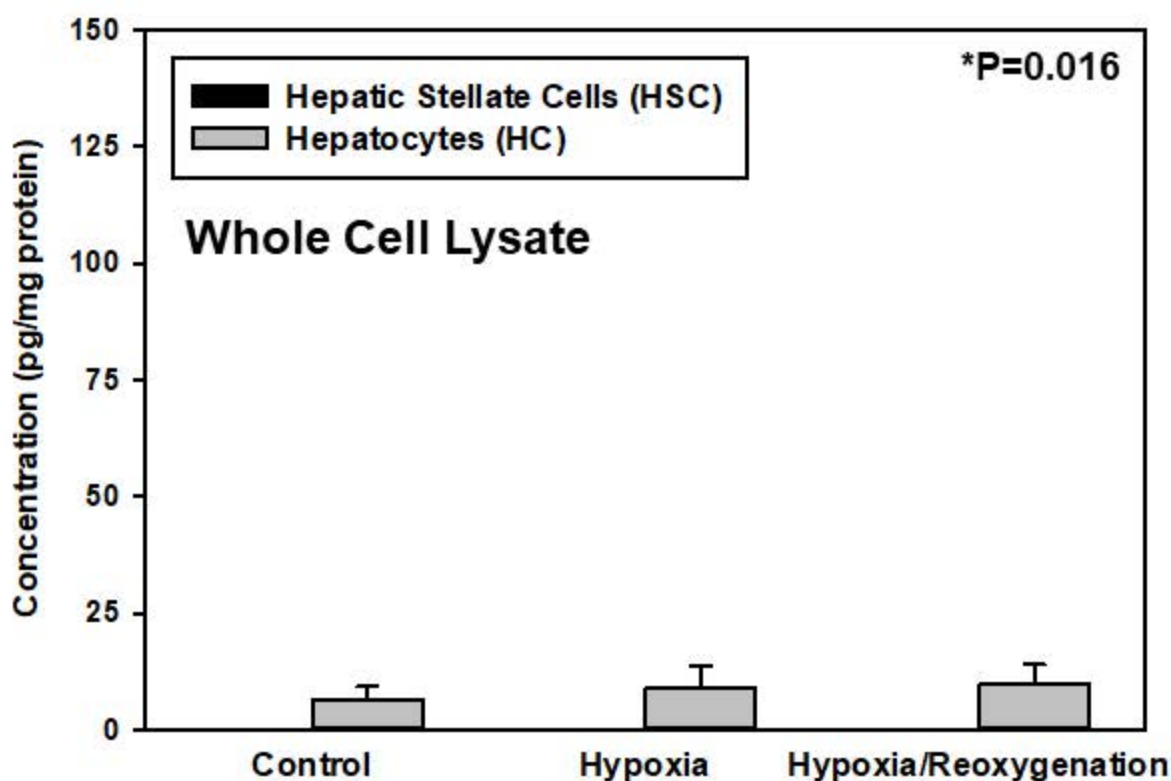

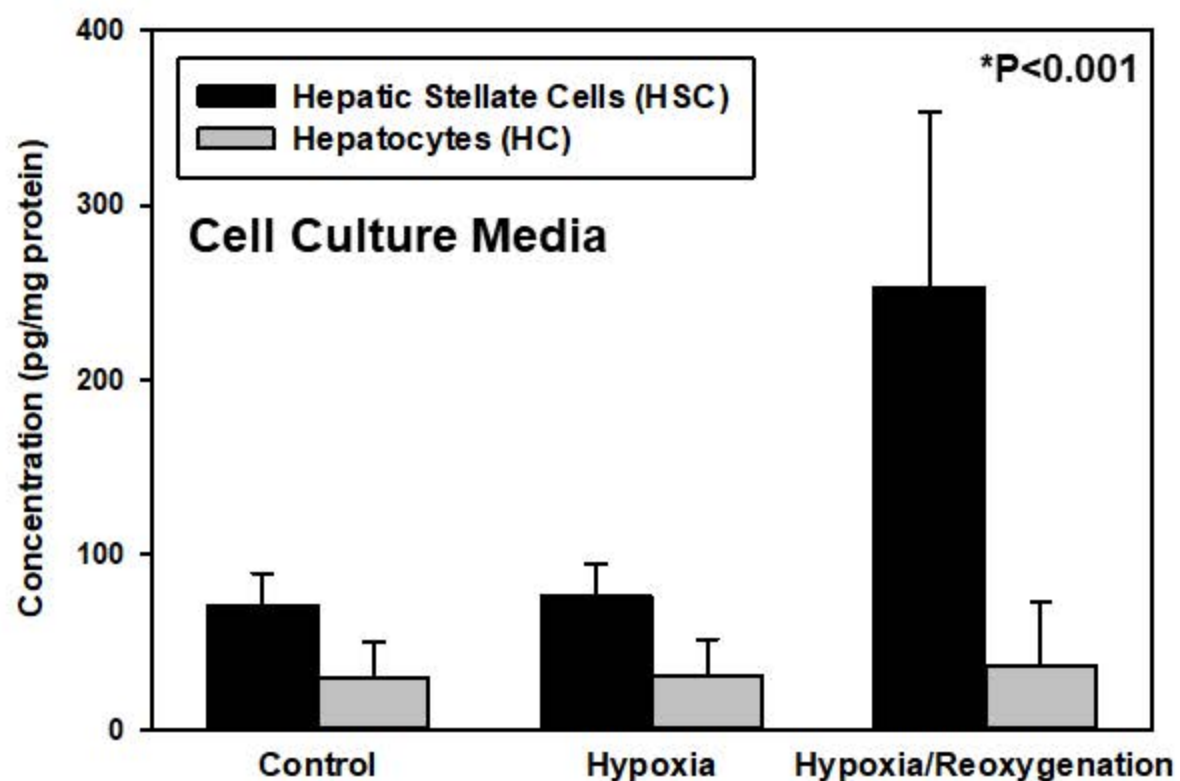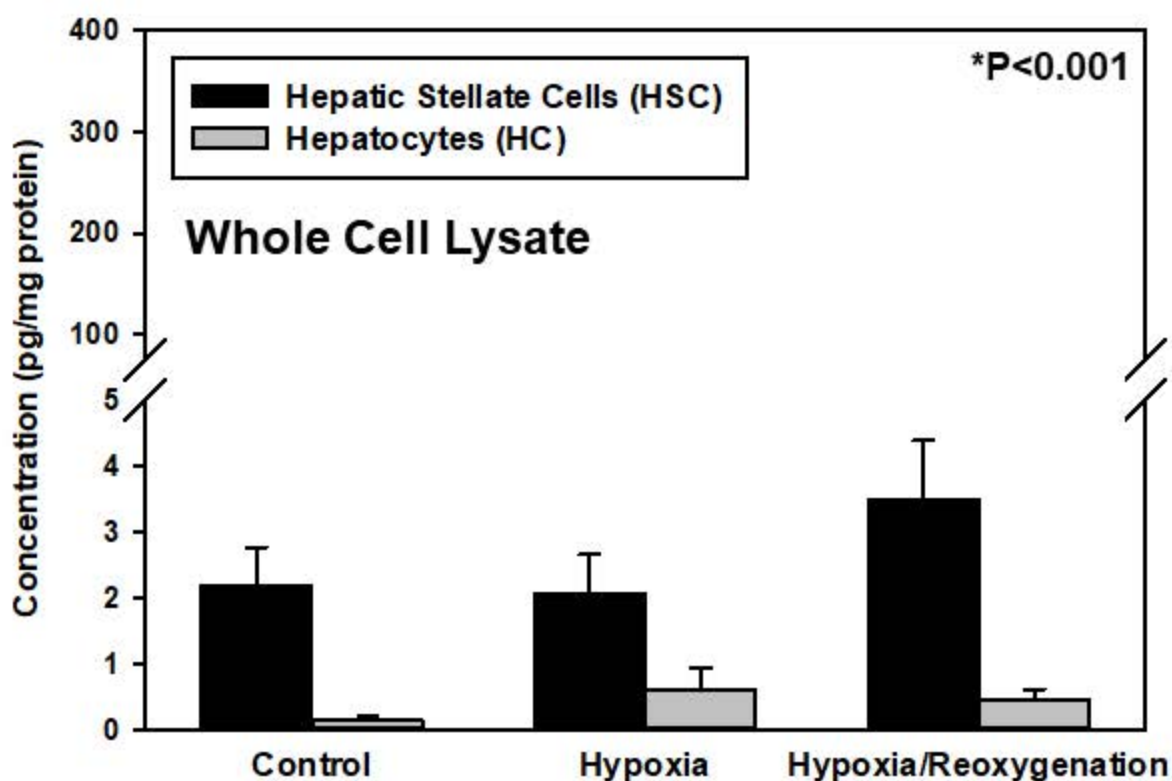

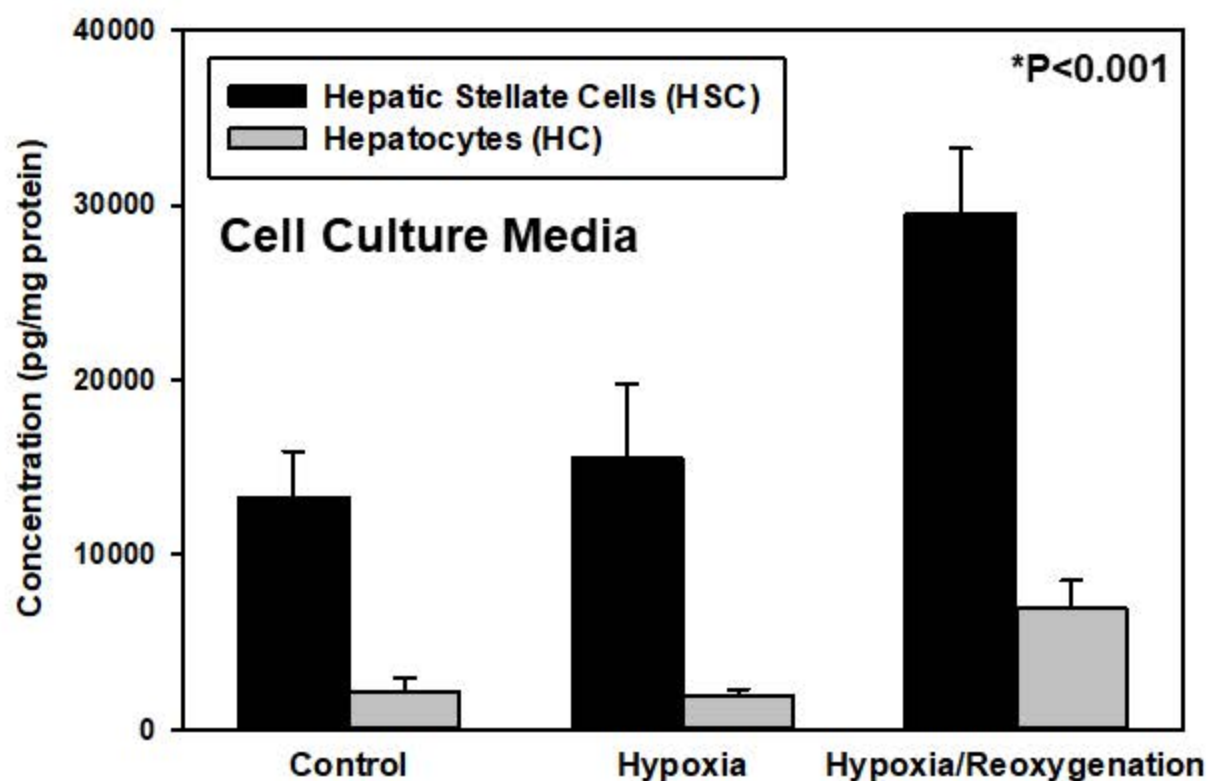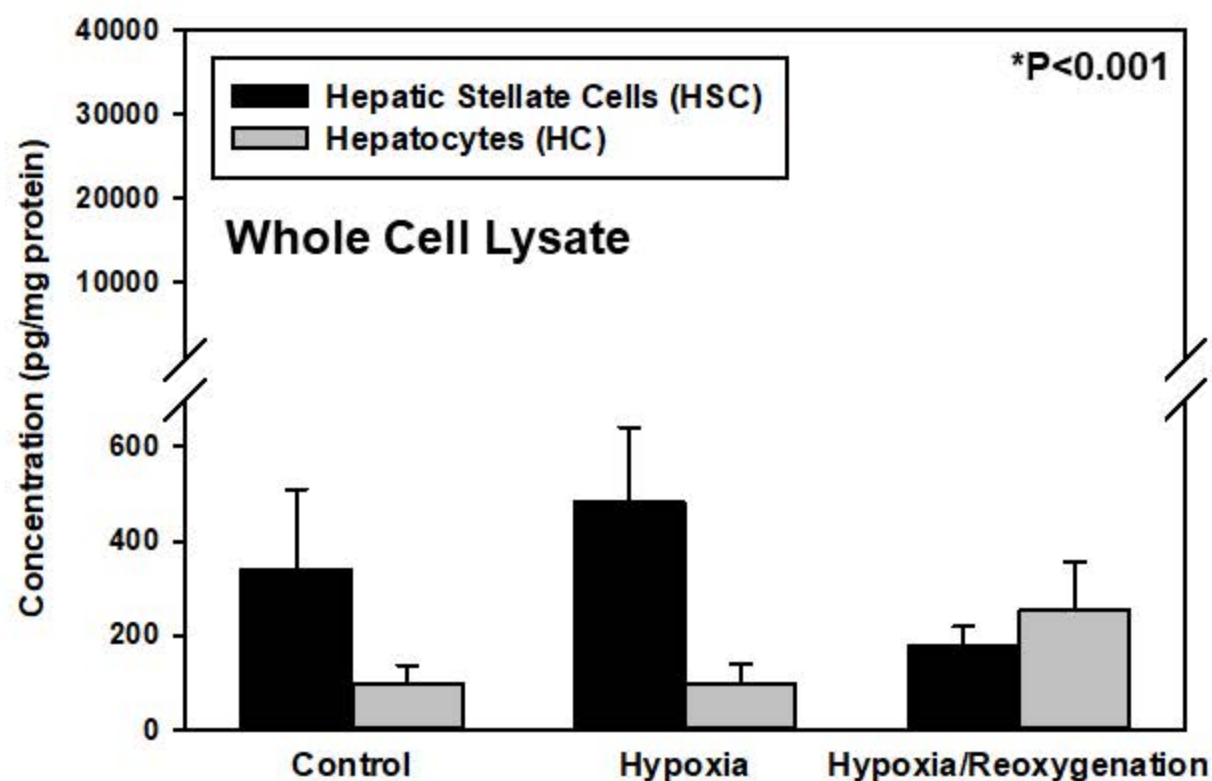

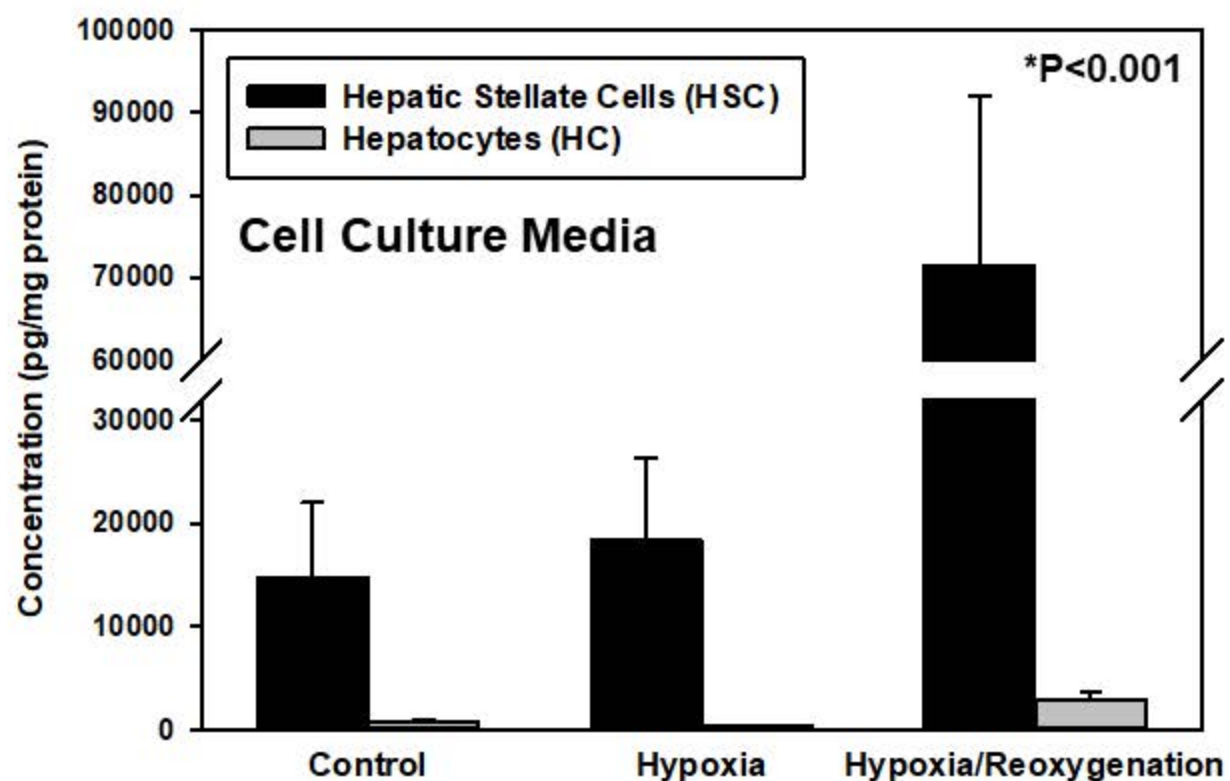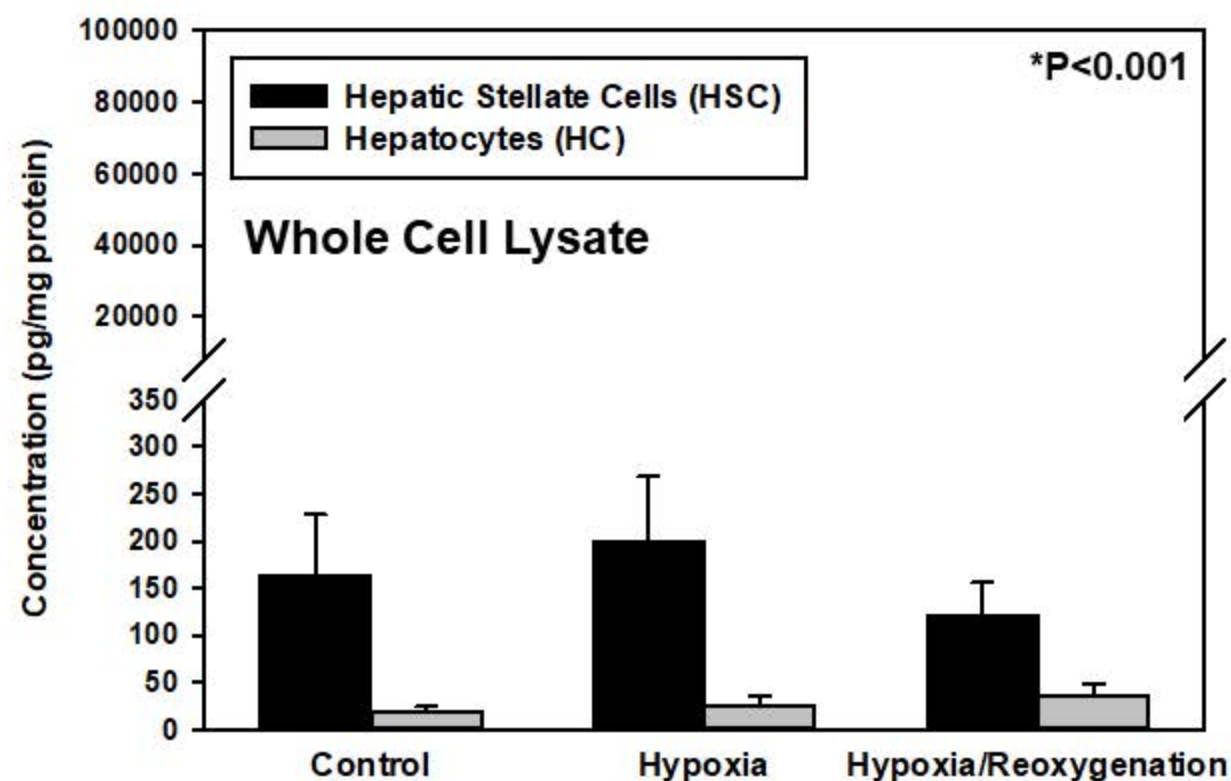

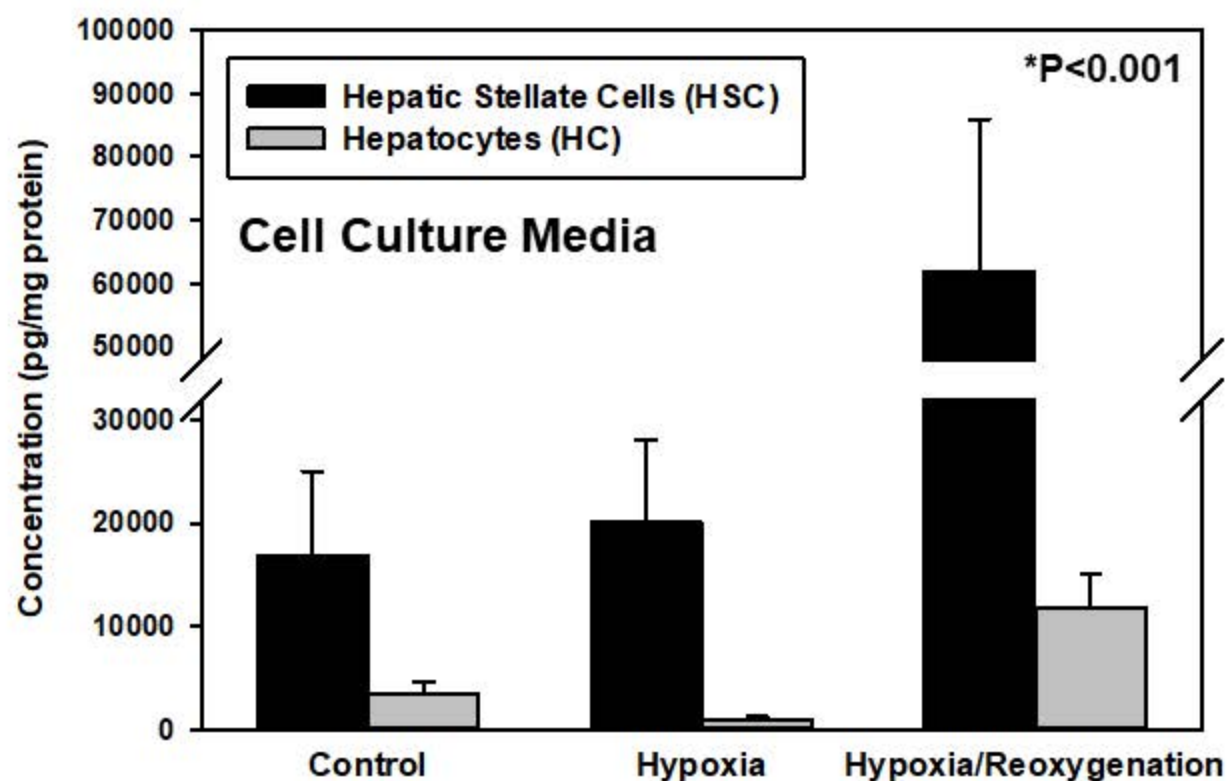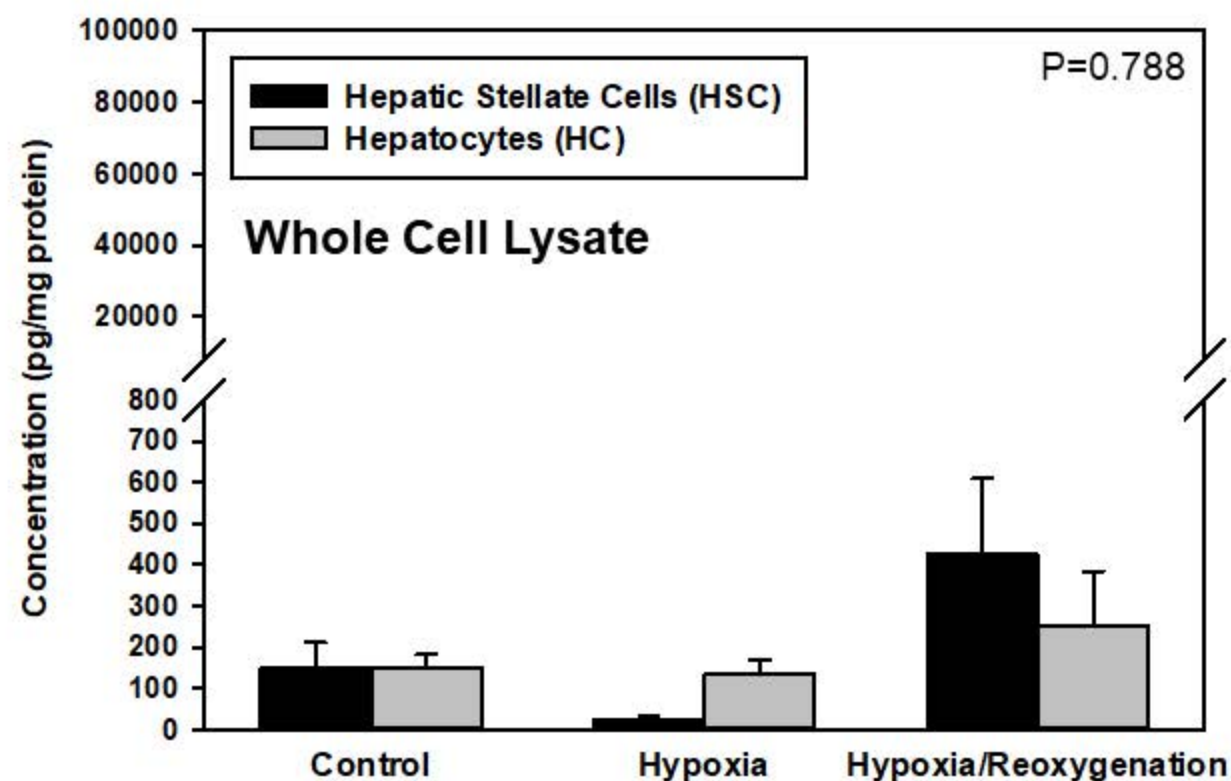

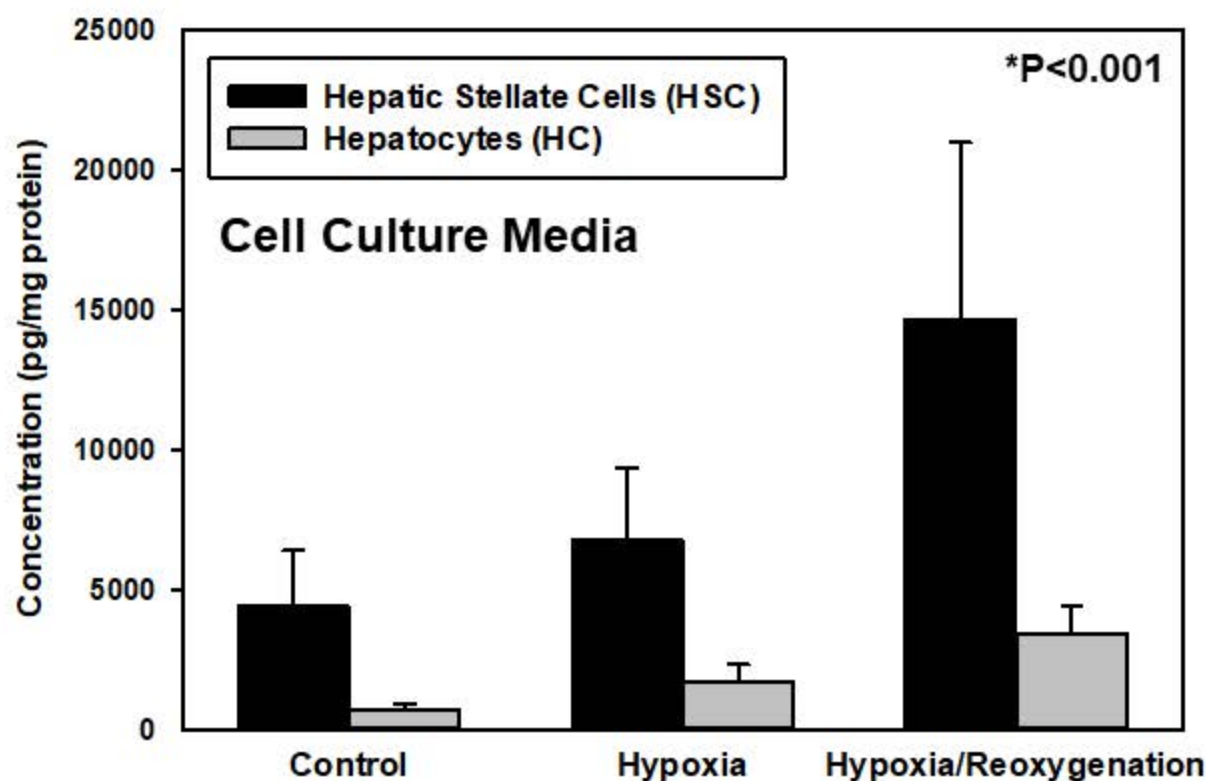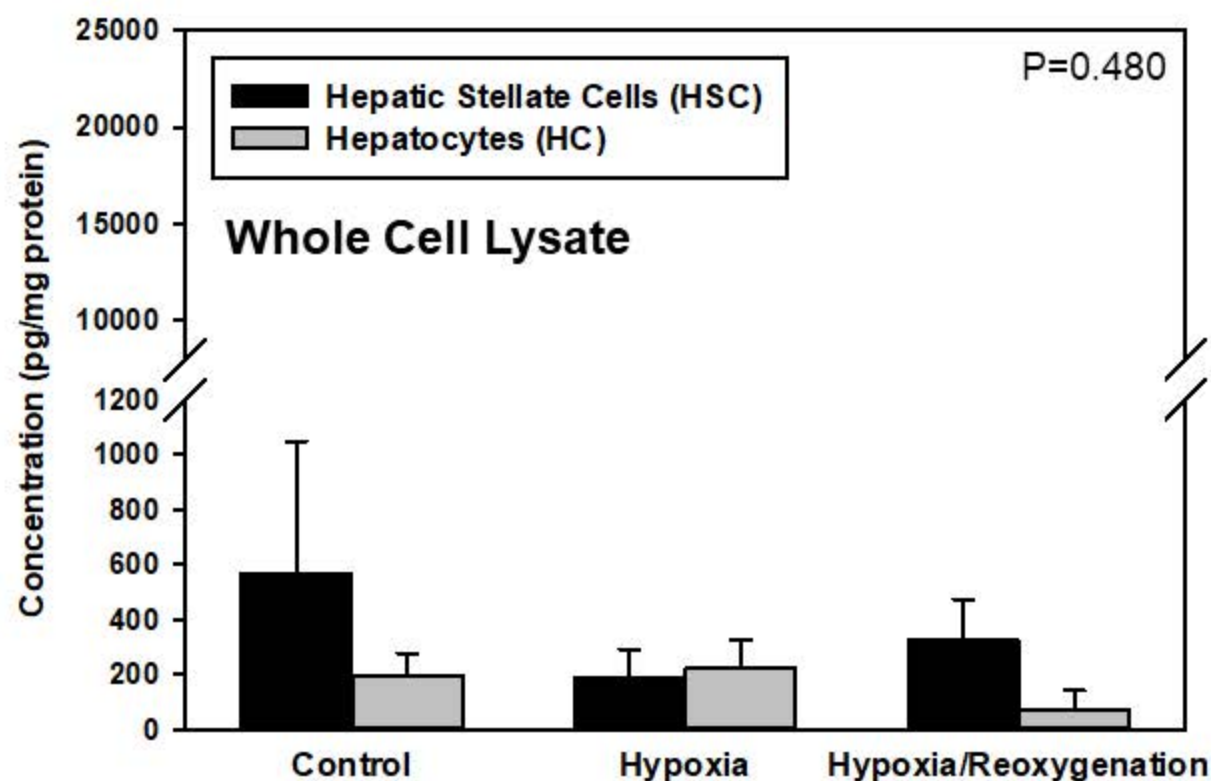

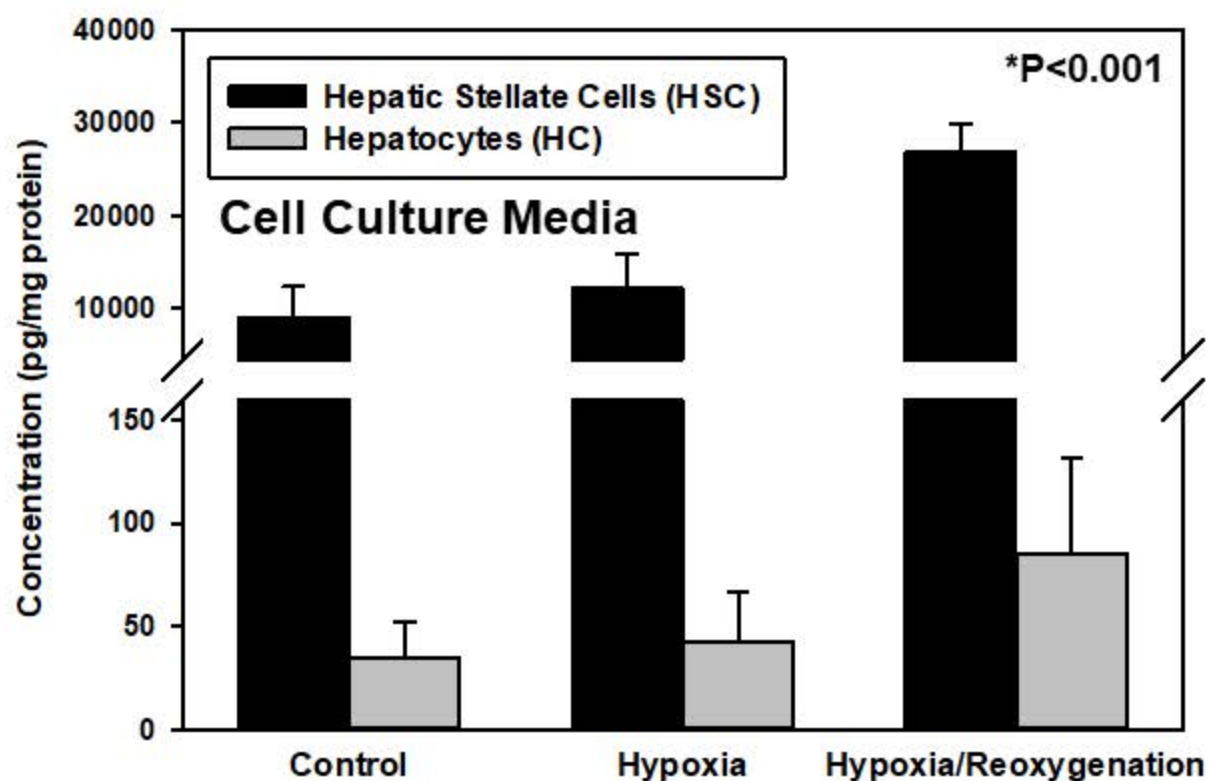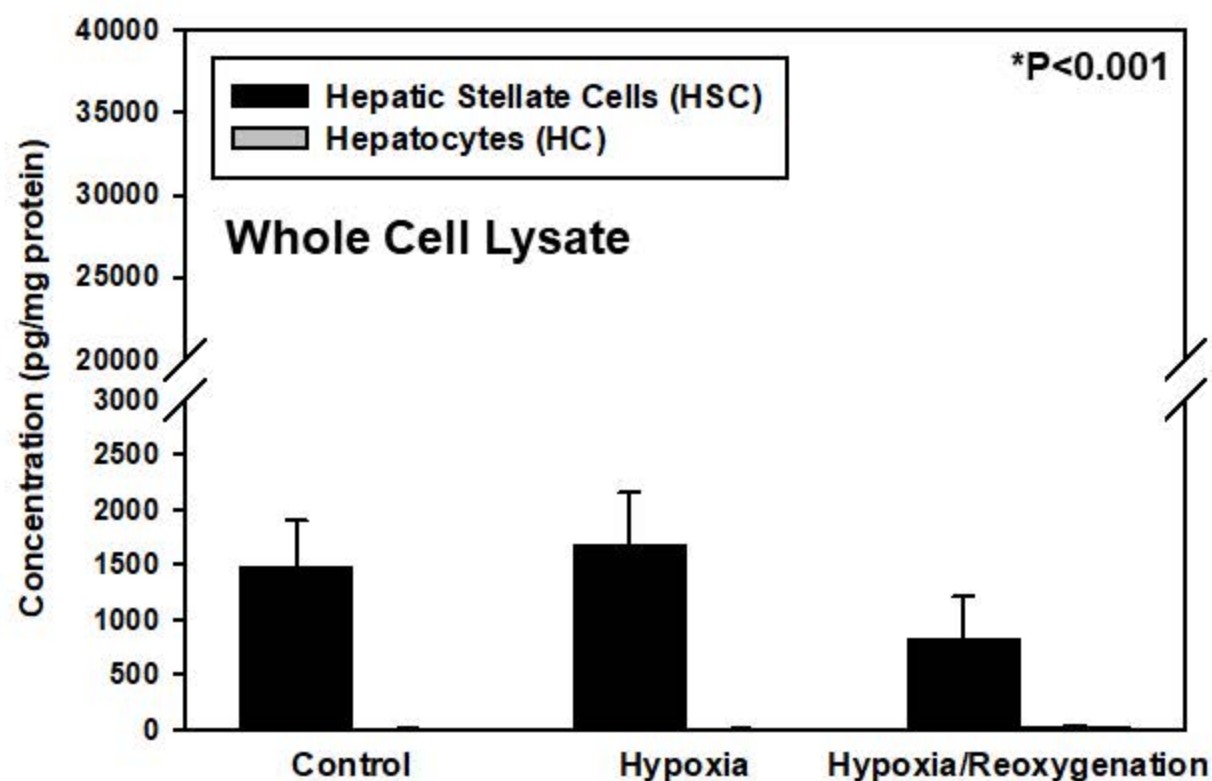

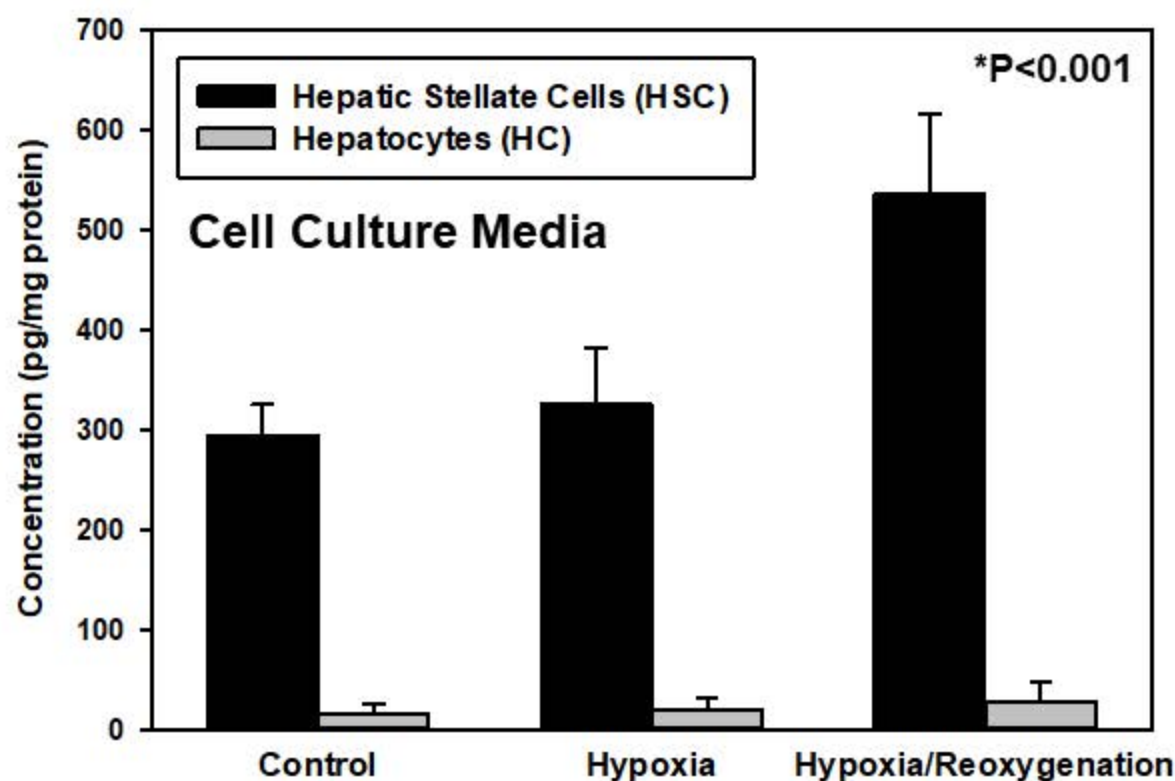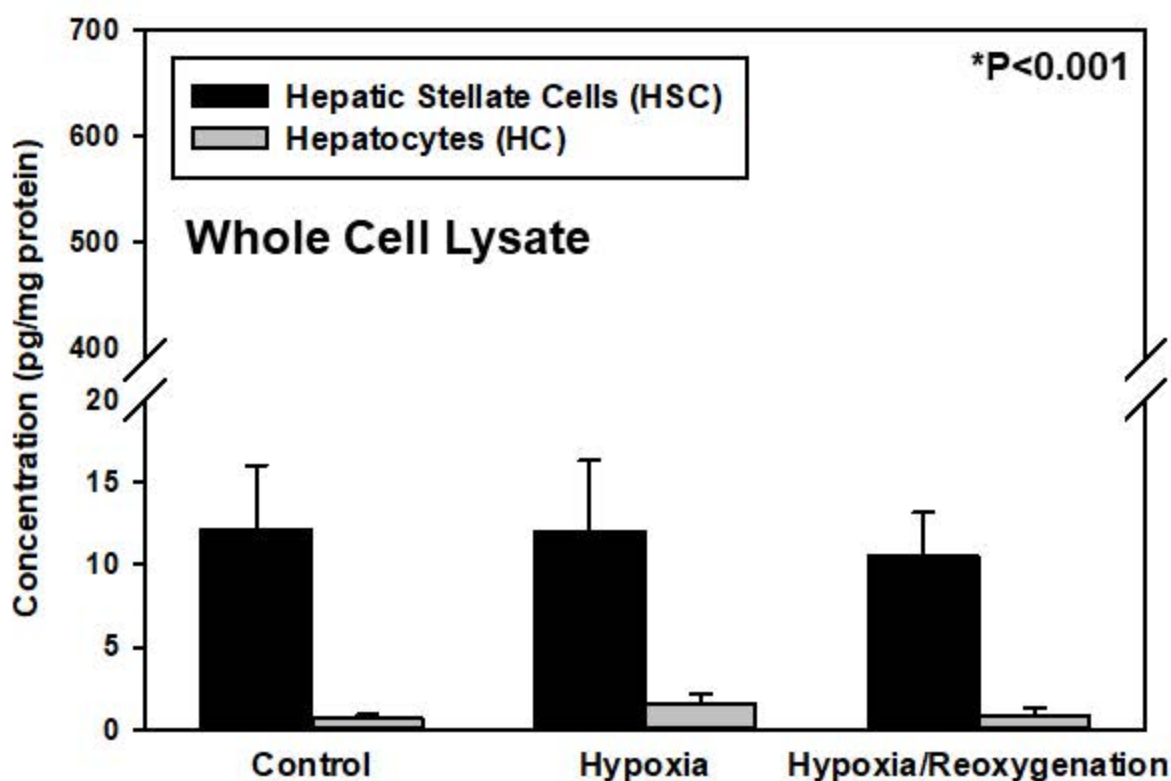

Suppl. Fig. 1

# VEGF

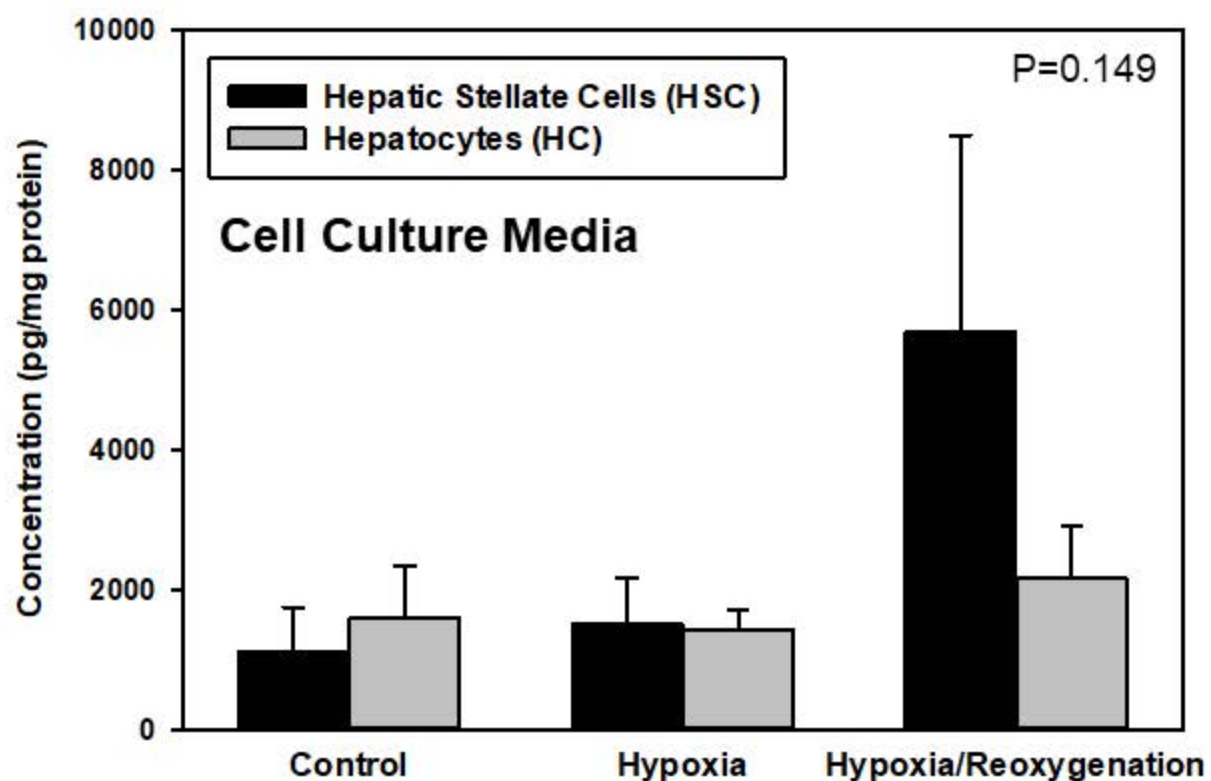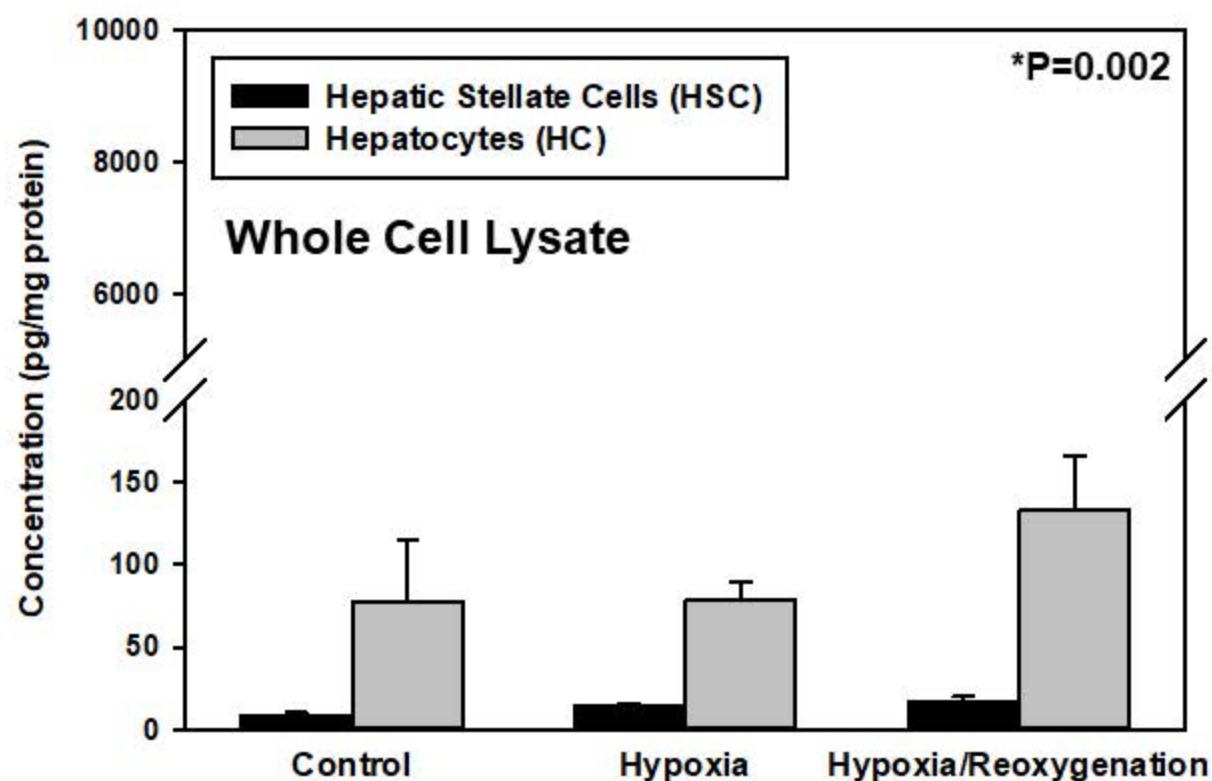

Supplement: Supplementary Figure 1 — Production of inflammatory mediators in mouse hepatic stellate cells (HSC) and hepatocytes (HC). Freshly isolated HSC and HC from C57BL/6 mice were cultured under 21% O2 for 6 h (control), hypoxia (1% O2) for 6 h or hypoxia (6 h) followed by reoxygenation for 18 h. Inflammatory mediators were measured by Luminex™ in both cell culture media and whole cell lysate as described in Materials and Methods. Results shown represent mean ± SEM (*P < 0.05, HSC vs. HC, analyzed by Two-Way ANOVA). [file Image1.PDF]
