# Supplementary material for: “Thinking” vs. “Talking”: Differential Autocrine Inflammatory Networks in Isolated Primary Hepatic Stellate Cells and Hepatocytes under Hypoxic Stress
Source: Front Physiol. 2017 Dec 22;8:1104. doi: 10.3389/fphys.2017.01104 (PMC5743931; doi:10.3389/fphys.2017.01104)
Supplement: Supplementary Figure 3 — Network Analysis of inflammatory mediators in mouse hepatic stellate cells (HSC) and hepatocytes (HC). Freshly isolated HSC and HC from C57BL/6 mice were cultured under 21% O2 for 24 h (control) or hypoxia (6 h) followed by reoxygenation for 18 h (as in Supplementary Table 1). Inflammatory mediators released into cell culture media (CCM) and in whole cell lysate (WCL) were measured by Luminex™ and Network Analysis was performed as described in Materials and Methods. Figure shows the networks (stringency level 0.95) for each experimental condition in both cell types in CCM (A) and WCL (B). Black and red arrows represent positive and negative connections, respectively. [file Image3.pdf]

Suppl. Fig. 3

**Cell Culture Media**

**A**

Control 24h

Hypoxia/Reoxygenation

Hepatic Stellate Cells

Hepatocytes

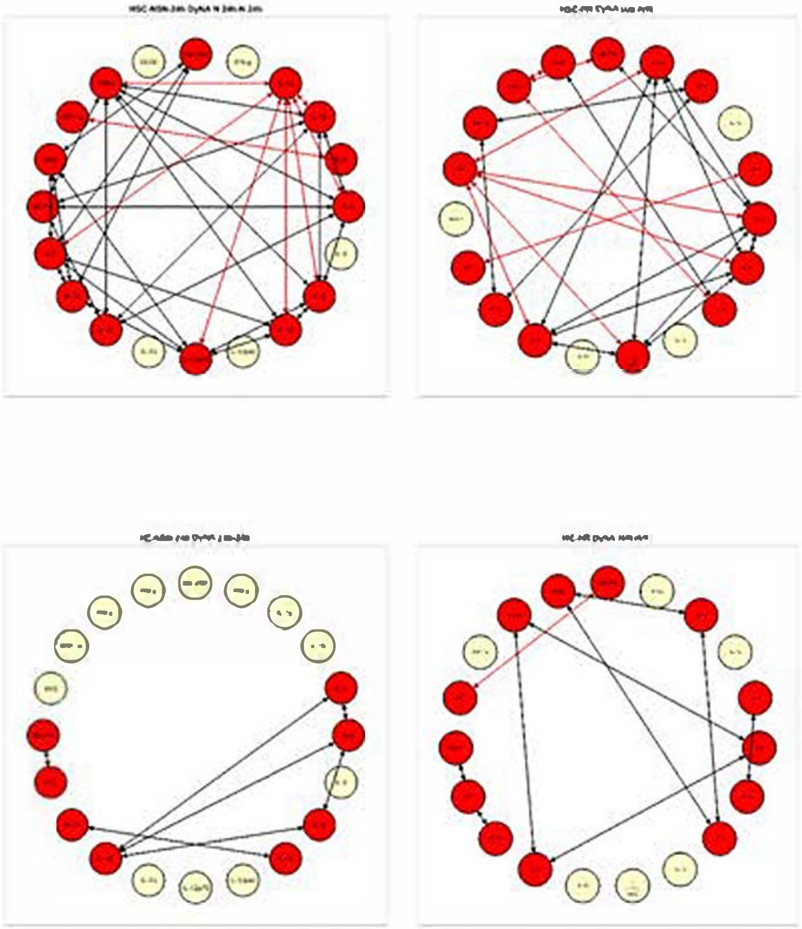

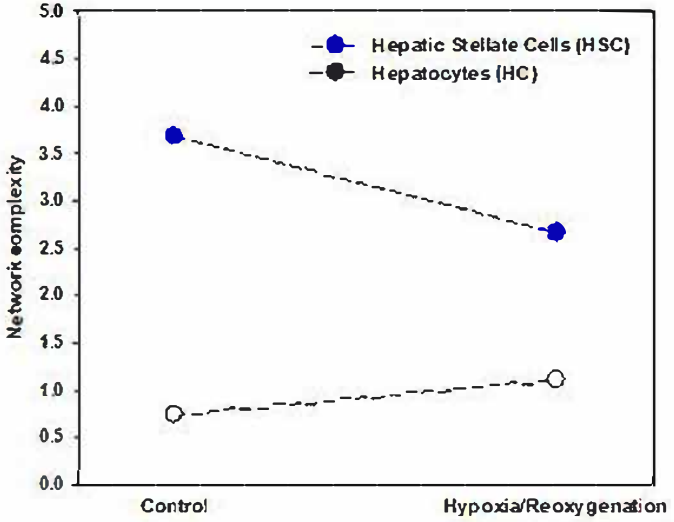


Stringency level 0.95

Suppl. Fig. 4

**Whole Cell Lysate**

**B**

Control 24h | Hypoxia/Reoxygenation

Hepatic Stellate Cells

Hepatocytes

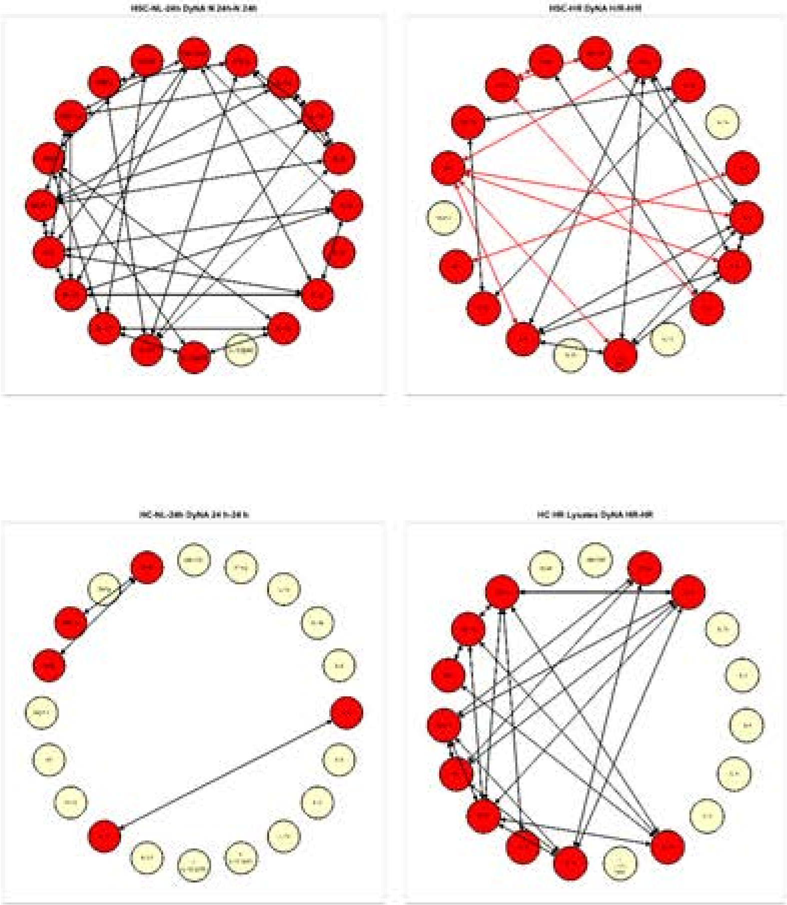

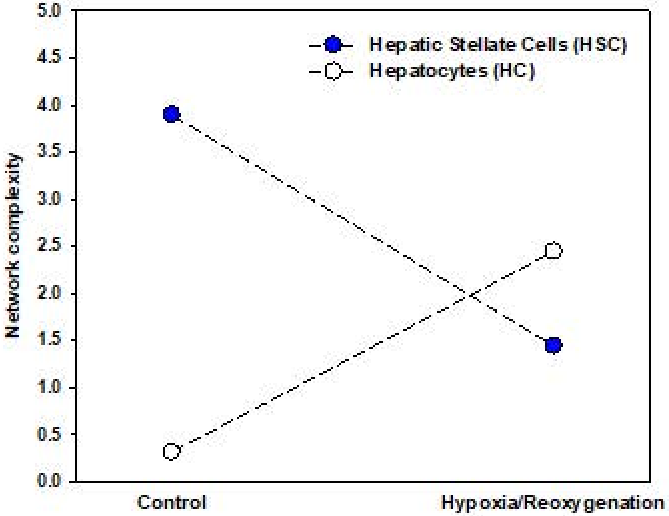


Stringency level 0.95
